# Global burden of disease study on COPD in the older adult: comprehensive analysis of environmental factors and interaction effects

**DOI:** 10.3389/fpubh.2025.1597793

**Published:** 2025-05-30

**Authors:** Yuwei Wang, Li Jin, Yuhui Dong, Erche Yang, Xiaoqun Niu, Jianchuan Mao, Chaoqun Yuan, Bo You, Yong Wang, Yanling Chai

**Affiliations:** ^1^Department of Respiratory and Critical Care Medicine, People’s Hospital of Yuechi County, Yuechi, China; ^2^Department of Anesthesiology, People’s Hospital of Yuechi County, Yuechi, China; ^3^Department of Anesthesiology, Bazhong Central Hospital, Bazhong, China; ^4^Department of Respiratory and Critical Care Medicine of Dali Bai Autonomous Prefecture People’s Hospital, Dali, China; ^5^Department of Respiratory and Critical Care Medicine, The Second Affiliated Hospital of Kunming Medical University, Kunming, China

**Keywords:** COPD (chronic obstructive pulmonary disease), older adult, environmental factors, air pollution, non-optimal temperature, global burden of disease (GBD)

## Abstract

**Background:**

Chronic obstructive pulmonary disease (COPD) is a leading global health issue, ranking fourth in mortality in 2021 per GBD 2021, with older adults most at risk due to aging-related vulnerabilities. Environmental factors like air pollution and temperature extremes are key contributors, with evidence suggesting their interaction worsens COPD. As the older adult population is projected to reach 2.1 billion by 2050, understanding these impacts is vital for public health.

**Method:**

Using GBD 2021 data, we analyzed COPD burden in older adults (1990–2021) due to air pollution and non-optimal temperature. Joinpoint regression and Bayesian models assessed trends in age-standardized mortality (ASMR) and disability-adjusted life year (ASDR) rates globally, regionally, and nationally. Spearman’s correlation evaluated socio-demographic index (SDI) associations, and standardized exposure value (SEV) data examined air pollution-temperature interactions.

**Result:**

Globally, most environmental COPD burdens declined or stabilized, but high temperature burdens rose (AAPC > 0, *p* < 0.05). By 2015, ambient PM2.5 overtook household air pollution as the top risk, with 2021 ASMR at 76.29 [54.62, 95.48] and ASDR at 1364.67 [981.69, 1687.82] per 100,000. Low- and middle-SDI regions, especially South and East Asia, faced the highest burdens, with rising PM2.5 and ozone impacts. Air pollution and temperature showed synergistic effects, with high temperature strongly correlating with increased PM2.5 and ozone exposure levels, amplifying COPD burden, except for household air pollution.

**Conclusion:**

This study reveals shifting COPD burdens, with ambient PM2.5 and high temperatures emerging as key challenges, particularly in lower-SDI areas. Synergistic air pollution-temperature effects highlight the need for integrated policies. These findings support targeted interventions to reduce COPD burden and enhance health equity in aging populations.

## 1 Introduction

Chronic obstructive pulmonary disease (COPD) is characterized by persistent respiratory symptoms and airflow limitation, typically resulting from airway and/or alveolar abnormalities caused by prolonged exposure to harmful particles or gases, encompassing conditions such as emphysema and chronic bronchitis (1, 2). According to the Global Burden of Disease (GBD) 2021 study, COPD was the fourth leading cause of death worldwide in 2021, accounting for over 3 million deaths and posing a significant challenge to global health systems (3). Older adults are particularly vulnerable to COPD due to physiological deterioration and declining immune function, making them the primary population bearing the disease burden (4). With the accelerating global trend of population aging, the number of older individuals continues to rise, rendering the health protection of this demographic a critical global priority. United Nations data project that by 2050, the global population aged 60 and above will reach 2.1 billion, comprising 22% of the total population (5). This profound demographic shift will exacerbate the burden of chronic diseases like COPD, placing increasing pressure on global public health systems. Aging not only elevates the incidence and mortality of COPD but also complicates its prevention, diagnosis, and treatment, underscoring the urgency of addressing this issue (6). Consequently, research focused on COPD in older adults is of paramount importance to tackle this growing public health challenge.

In recent years, the role of environmental factors in the COPD disease burden has garnered widespread attention, with air pollution and temperature variations emerging as key research themes. Air pollution can induce and exacerbate COPD through mechanisms such as inflammation, oxidative stress, airway hyperresponsiveness, and immune damage (7). High and low temperatures pose threats to COPD patients by directly irritating the airways, intensifying inflammation, and increasing infection risks (8). As industrialization accelerates globally, air pollution has become increasingly severe, with ambient particulate matter (e.g., PM2.5), household air pollution, ozone, and nitrogen dioxide significantly contributing to COPD onset and progression (9). Concurrently, global climate change has led to more frequent extreme temperature events, with a clear trend of global warming, posing additional health risks to COPD patients, particularly older adults (10). Notably, previous studies have identified a synergistic interaction between temperature and air pollution, collectively amplifying the COPD burden (11–13). For instance, during high temperatures, ozone concentrations rise significantly, enhancing the toxicity of particles like PM2.5, which more readily penetrate the alveoli, intensifying inflammation and oxidative stress (14). High temperatures also stabilize atmospheric stratification, reducing pollutant dispersion and increasing near-surface particle accumulation, thereby elevating patient exposure levels. Conversely, in low temperatures, cold air increases airway mucosal vulnerability and sensitivity to pollutants, with PM2.5 and NO₂ exhibiting heightened irritative effects, potentially triggering airway spasms and inflammatory responses (15). However, there remains a lack of global-scale simulation studies to further validate and quantify these findings.

Given the intensifying global aging trend and escalating environmental challenges, research on the COPD burden in older adults is increasingly critical. This study leverages the GBD 2021 database, employing Joinpoint regression and Bayesian simulation methods, to systematically evaluate trends in COPD burden among older adults attributable to air pollution and non-optimal temperature from 1990 to 2021 across global, regional, and national levels, with a primary focus on analyzing their synergistic interactions. We aim to elucidate burden patterns, uncover mechanisms driving these environmental effects, and provide a scientific basis for targeted prevention and intervention strategies, offering actionable guidance for global health policymakers.

## 2 Method

### 2.1 Data sources and measurement of disease burden and trends

Data on the COPD burden among older adults and corresponding environmental factors from 1990 to 2021 were obtained from the Global Health Data Exchange (GHDx) online platform (https://vizhub.healthdata.org/gbd-results/). These data were sourced from the GBD 2021 project, which quantifies disease burden using disability-adjusted life years (DALYs) and provides multi-scale observations, including attributable effects of 87 risk factors, with detailed methodologies previously described (16, 17). The Socio-Demographic Index (SDI), a composite indicator proposed by the GBD project, comprises per capita expected years of education, total fertility rate, and per capita income, serving as a measure of a country or region’s social and demographic development (18). In this study, we quantified the burden in older adults using age-standardized rates, calculating the age-standardized mortality rate (ASMR) and age-standardized DALY rate (ASDR) via Equation 1:


(1) $$\frac{\sum_{i=1}^{i}w_{i}x_{i}}{\sum_{i=1}^{i}w_{i}}$$

where iii represents the i-th age group, $w_{i}$ denotes the population of the i-th age group in the standard population, and $x_{i}$​ indicates the disease burden for the i-th age group.

In the GBD database, COPD is defined as a condition characterized by persistent respiratory symptoms and airflow limitation, typically resulting from airway and/or alveolar abnormalities due to prolonged exposure to harmful particles or gases, including emphysema and chronic bronchitis. This study included environmental factors such as air pollution and non-optimal temperature, along with their sub-risk factors. Air pollution encompasses ambient particulate matter pollution (PM2.5, defined as the annual mean mass concentration of particles with an aerodynamic diameter less than 2.5 μm per cubic meter, population-weighted, with a theoretical minimum risk exposure level [TMREL] uniformly distributed between 2.4 and 5.9 μg/m^3^), household air pollution (HAP, exposure to particles less than 2.5 μm in diameter from cooking with solid fuels such as coal, charcoal, wood, agricultural residues, and animal dung), ambient ozone pollution (defined as the seasonal maximum daily 8-h average ozone concentration, with a TMREL uniformly distributed between 29.1 and 35.7 parts per billion), and nitrogen dioxide pollution (associated with traffic and industrial emissions, TMREL not specified). Non-optimal temperature is defined as the combined burden of high and low temperatures, with effects measured as deviations above and below the TMREL (population-weighted mean of 25.6°C) (19). The TMREL represents the theoretical exposure level at which health risks are minimized, and deviations from this level increase the disease burden. The population attributable fraction (PAF) quantifies the proportion of the disease burden attributable to such deviations, estimating the percentage reduction in burden if exposure were reduced to the TMREL, calculated using Equation 2:


(2) $$\text{PAF}=\frac{\int \text{RR}(x)P(x)\text{dx}-\int \text{RR}(x)P_{\text{TMREL}}(x)\text{dx}}{\int \text{RR}(x)P(x)\text{dx}}$$

where RR(x) is the relative risk at exposure level x, P(x) is the population distribution at exposure level x, and $P_{\text{TMREL}}(x)$ is the population distribution at the TMREL.

Notably, the dataset used in this study is de-identified, and the GBD project has been approved by the Institutional Review Board of the University of Washington, with informed consent waived, thus requiring no additional ethical approval.

### 2.2 Statistical analysis

All rates in this study are expressed per 100,000 population, with burden levels quantified using age-standardized rates. As GBD data are statistically computed using Bayesian methods, all indicators are presented with 95% uncertainty intervals (95% UI). In regions with limited data, wider UI ranges indicate reduced precision. To examine trends in the COPD burden among older adults attributable to various risk factors, we used Joinpoint software, developed by the National Cancer Institute (NCI), to calculate the average annual percent change (AAPC) over the study period. Given the study’s temporal scope, we set the minimum number of joinpoints to 0 and the maximum to 5 to ensure segment stability. The model assumes linear trends on a logarithmic scale and employs heteroscedasticity-consistent standard error estimation to account for potential variability in health data. Optimal joinpoint numbers were identified through an iterative process, with the annual percent change (APC) for each segment calculated using Equation 3. A permutation test with an overall Type I error rate of 0.05 and 1,000 Monte Carlo simulations was used to assess whether additional joinpoints significantly improved model fit. The final model selected the fit with the fewest joinpoints supported by the data. The AAPC, summarizing the overall trend across the study period, was calculated using Equation 4:


(3) $$\begin{array}{c} \text{APC}=100\times (\exp(\beta )-1) \end{array}$$

(4) $$\begin{array}{c} \text{AAPC}=100\times (\exp(\frac{\sum_{i=1}^{i}w_{i}\beta_{i}}{\sum_{i=1}^{i}w_{i}})-1) \end{array}$$

where i is the total number of segments, β is the regression slope on the logarithmic scale for each segment, and $w_{i}$ is the number of years in each segment for weighted calculation.

To further explore the relationship between the COPD burden in older adults and social development levels, we linked SDI data with disease burden data by region, using Spearman’s correlation analysis to assess the association between SDI levels and COPD burden. This approach was extended to investigate potential synergistic effects between the two main risk factor categories, examining correlations between air pollution exposure levels and the COPD burden due to non-optimal temperature, and vice versa. The Summary Exposure Value (SEV), a GBD metric, was used to quantify population-weighted exposure levels to these factors, normalized on a 0–100 scale, enabling analysis of their individual and synergistic impacts.

All analyses were conducted using R software (version 4.3.2) and Joinpoint software (version 4.9.1). Data cleaning and processing were performed with the tidyverse and dplyr packages, while data visualization was conducted using the ggplot2 package. All statistical tests were two-sided, with a *p*-value < 0.05 considered statistically significant.

## 3 Result

### 3.1 Trends and burden at global and regional levels

**Figure 1:**
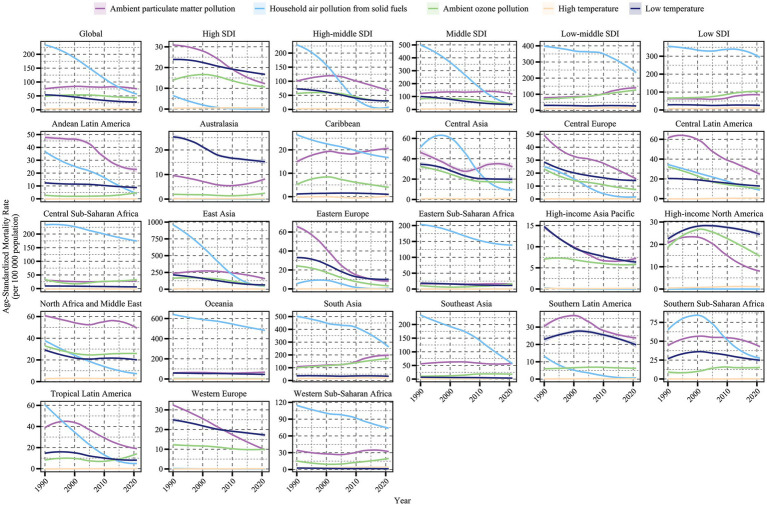
Trends in age-standardized mortality rates of COPD attributable to environmental factors among older adults by region and SDI level, 1990–2021.

**Figure 2:**
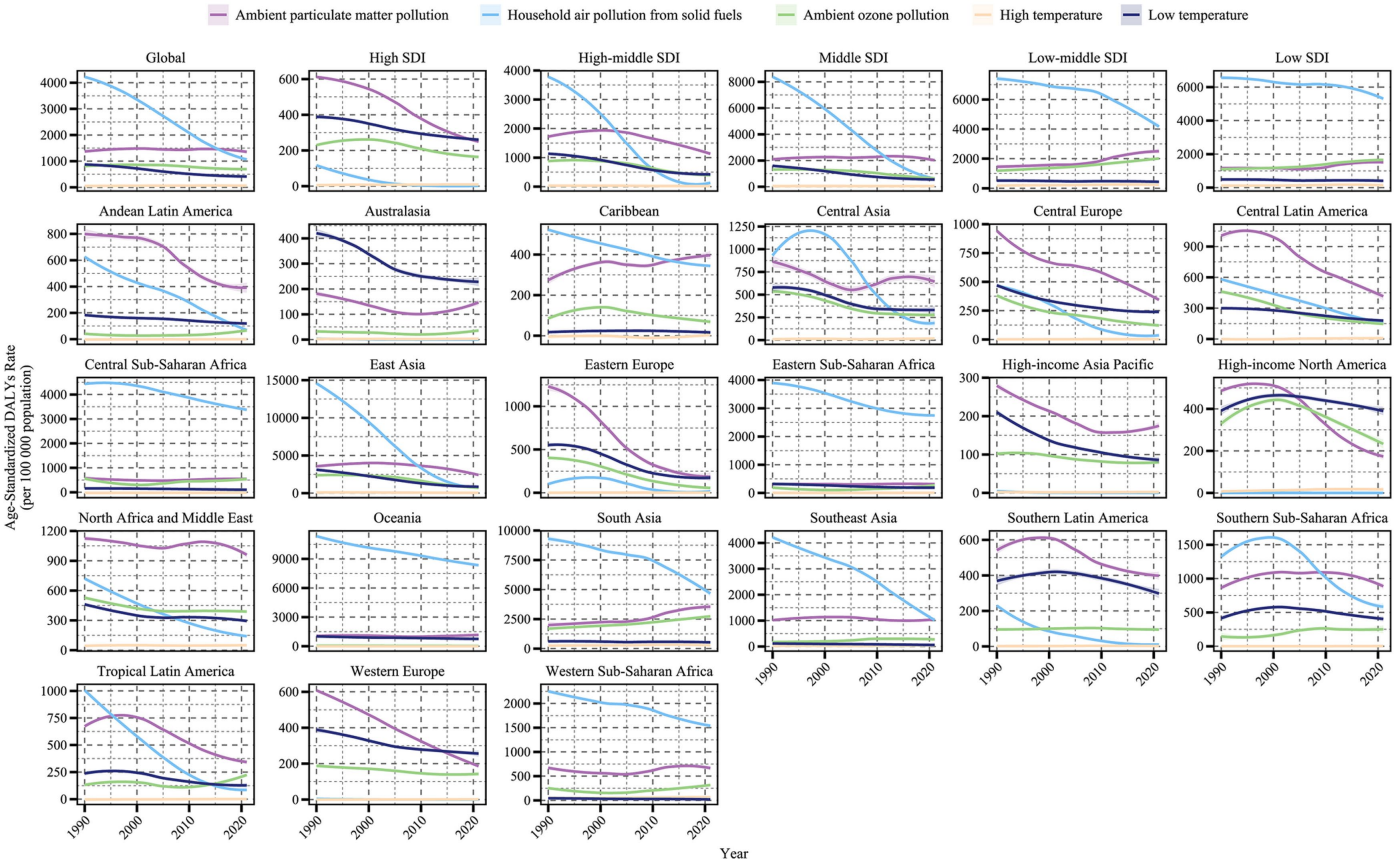
Trends in age-standardized DALY rates of COPD attributable to environmental factors among older adults by region and SDI level, 1990–2021.

A comprehensive assessment of COPD attributable to air pollution and non-optimal temperature risk factors revealed trends in COPD burden from 1990 to 2021 for each risk factor (Figures 1, 2). Additionally, we quantified the ASMR and ASDR levels of COPD in older adults due to these environmental factors in 1990 and 2021, along with their overall change (AAPC), with detailed values presented in Tables 1, 2. Globally, the COPD burden from most environmental factors either declined or remained stable over the past few decades, except for high temperature, which showed a significant increase in both mortality and DALYs (AAPC > 0, *p* < 0.05). Among these environmental risk factors, household air pollution from solid fuels was historically the leading contributor to COPD burden, far surpassing other factors. However, this pattern shifted as the burden from household air pollution decreased, and by 2015, ambient particulate matter pollution surpassed it to become the leading global environmental contributor to COPD burden. In 2021, the global ASMR for COPD in older adults due to ambient particulate matter pollution was 76.29 [54.62, 95.48] per 100,000 population, with an ASDR of 1364.67 [981.69, 1687.82] per 100,000 population. Household air pollution from solid fuels resulted in an ASMR of 60.28 [34.89, 104.48] per 100,000 population and an ASDR of 1134.03 [675.61, 1903.06] per 100,000 population. Ambient ozone pollution contributed to an ASMR of 43.78 [9.57, 75.25] per 100,000 population and an ASDR of 694.12 [151.79, 1190.36] per 100,000 population. Low temperature led to an ASMR of 27.8 [21.99, 33.89] per 100,000 population and an ASDR of 411.38 [324.46, 504.24] per 100,000 population, while high temperature resulted in an ASMR of 3.27 [−0.71, 9.12] per 100,000 population and an ASDR of 53.3 [−10.54, 146.6] per 100,000 population.

**Table 1:** Age-standardized mortality rates (ASMR) and average annual percent change (AAPC) of COPD in older adults attributable to environmental factors across global, SDI, and GBD regions, 1990–2021.

	Ambient particulate matter pollution			Household air pollution from solid fuels			Ambient ozone pollution			Low temperature			High temperature		
Location	1990	2021	AAPC	1990	2021	AAPC	1990	2021	AAPC	1990	2021	AAPC	1990	2021	AAPC
Global	76.73[50.23,113.55]	76.29[54.62,95.48]	−0.21[−0.65,0.24]	231.41[180.7,277.41]	60.28[34.89,104.48]	−4.3[−4.56, –4.05]	49.16[10.71,86.42]	43.78[9.57,75.25]	−0.3[−0.77,0.18]	51.68[43.19,60.42]	27.8[21.99,33.89]	−2.09[−2.94,–1.23]	2.88[−1.37,9.57]	3.27[−0.71,9.12]	0.92[0.42,1.42]
High SDI	31.21[20.23,44.74]	12.59[8.71,16.74]	−2.92[−3.27,–2.58]	6.34[2.38,13.08]	0.04[0,0.38]	−14.93[−15.38,–14.47]	14.69[3.16,25.53]	10.81[2.36,18.79]	−0.93[−1.48,–0.38]	23[20.14,25.36]	16.93[14.55,18.7]	−1.29[−1.41,–1.16]	0.38[−0.81,1.93]	0.5[−0.63,2.08]	1.33[0.61,2.06]
High-middle SDI	103.04[63.47,159.21]	71.05[52.95,89.62]	−1.25[−1.5,–1]	226.78[160.86,288.7]	5.35[0.2,33.18]	−11.64[−12.34,–10.93]	56.83[12.02,99.17]	28.95[5.96,51.14]	−2.1[−2.65,–1.55]	70.06[59.35,80.4]	30.5[24.79,36.47]	−2.7[−3.48,–1.92]	2.31[−1.98,8.84]	1.44[−1.34,5.31]	−1.64[−2.32,–0.95]
Middle SDI	124.65[72.05,200.41]	123.16[86.14,154.54]	−0.24[−0.98,0.5]	495.77[387.63,590.62]	47.07[9.37,134.95]	−7.44[−8.2,–6.68]	83.08[17.79,145.1]	44.85[9.84,77.94]	−2.03[−2.74,–1.3]	96.8[79.58,114.16]	39.54[31.26,48.73]	−3.07[−4.19,–1.95]	3.26[−2.67,11.72]	3.03[−0.72,8.35]	−0.1[−0.68,0.48]
Low-middle SDI	78.73[46.12,121.2]	134.16[77.84,186.66]	1.71[0.86,2.56]	392.03[302.22,490.53]	247.43[158.29,352.3]	−1.48[−1.8,–1.15]	69.5[13.55,126.74]	123.64[26.77,210.14]	1.96[1.21,2.72]	29.68[15.05,47.97]	26.06[12.01,42.23]	−0.3[−0.6,0]	9.48[−1.01,26.15]	12.03[−0.67,29.77]	1.42[0.84,2]
Low SDI	63.12[36.98,96.85]	80.42[51.56,115]	0.65[−0.01,1.32]	344.7[262.51,431.88]	290.86[225.54,355.79]	−0.53[−1.01,–0.04]	64.88[12.49,118.7]	101.15[21.43,174.22]	1.51[0.7,2.33]	27.46[15.87,41.25]	24.46[13.81,36.72]	−0.63[−2.46,1.24]	5.56[−1.36,16.9]	7.11[−1.41,19.6]	0.25[−2.56,3.14]
Western Europe	32.81[15.21,56.82]	10.28[6.88,13.87]	−3.76[−4,–3.51]	0.21[0,1.77]	0.01[0,0.06]	−10.34[−10.53,–10.16]	12.72[2.8,22.3]	9.76[2.14,17.06]	−0.76[−2.1,0.59]	23.77[20.62,27.14]	17.52[14.88,20.07]	−1.25[−1.41,–1.08]	0.02[−0.48,0.5]	0.08[−0.26,0.46]	NS
Central Europe	49.58[27.34,77.95]	16.87[13.03,21.09]	−3.45[−3.86,–3.04]	24.72[6.42,73.1]	1.42[0.04,10.47]	−8.93[−9.61,–8.23]	22.34[4.89,39.41]	7.41[1.6,13.24]	−3.82[−5.1,–2.51]	27.79[24.39,31.37]	15.22[13.15,17.15]	−2.32[−2.67,–1.96]	0.05[−1.01,1.25]	0.26[−0.63,1.37]	NS
Eastern Europe	63.26[28.63,103.67]	8.08[4.96,11.74]	−6.61[−7.29,–5.93]	6.29[1.46,24.46]	0.39[0.05,2.04]	−8.62[−9.58,–7.64]	23.31[4.81,41.53]	3.16[0.65,5.77]	−6.24[−6.94,–5.54]	31.26[27.55,33.54]	9.78[8.16,11.24]	−3.81[−4.72,–2.9]	−0.04[−0.66,0.54]	0.13[−0.32,0.57]	NS
East Asia	239.34[112.27,432.1]	165.28[117.14,212.43]	−1.24[−1.51,–0.98]	950.38[717,1157.29]	52.1[13.13,159.77]	−9.04[−9.99,–8.09]	165.39[34.74,290]	55.27[11.45,98.7]	−3.57[−4.15,–2.98]	206.01[167.91,241.78]	64.02[50.85,78.64]	−3.92[−5.09,–2.74]	7.07[−6.22,26.71]	3.08[−2.41,10.76]	−2.81[−3.6,–2.01]
Central Asia	43.61[19.87,80.94]	31.69[22.23,41.77]	−1.03[−2.18,0.14]	51.81[23.02,99.31]	9.37[4.42,19.97]	−5.31[−5.81,–4.81]	32.41[6.45,56.58]	16[3.07,29.56]	−1.99[−2.75,–1.22]	32.5[27.91,36.14]	19.02[16.2,21.93]	−1.7[−2.84,–0.54]	0.77[−1.32,3.03]	1.19[−0.65,3.25]	1.83[−1.05,4.78]
South Asia	107.18[56.32,174.5]	188.8[120.2,252.56]	1.7[0.71,2.7]	490.02[369.02,627.14]	275.93[180.12,393.83]	−1.75[−2.2,–1.3]	99.83[19.17,182.46]	167.6[36.29,284.79]	1.76[0.96,2.58]	34.93[13.67,61.09]	31.48[12.02,52.82]	−0.2[−0.57,0.16]	13.07[−2.24,36.54]	14.92[−1.66,37.69]	0.02[−2.35,2.44]
Southeast Asia	55.36[24.36,100.09]	57.18[38.37,76.26]	0.07[−0.09,0.23]	235.09[170.01,297.88]	66.67[27.1,125.56]	−4.05[−4.15,–3.95]	9.02[1.7,18.06]	19.89[3.97,36.29]	2.6[−1.32,6.68]	6.32[3.07,10.05]	4.04[2.15,6.07]	−1.9[−2.31,–1.48]	1.19[−0.3,2.63]	2.18[1.06,4.11]	NS
High-income North America	21.18[7.62,38.35]	8[3.77,12.83]	−2.94[−3.56,–2.33]	0.02[0,0.12]	0[0,0.02]	−5.08[−5.42,–4.74]	20.66[4.55,35.79]	15.23[3.29,26.62]	−0.86[−1.27,–0.44]	22.22[18.79,24.52]	24.48[20.55,27.26]	0.26[−0.03,0.56]	0.46[−1.52,2.86]	1.03[−1.35,4.55]	2.83[1.69,3.98]
Tropical Latin America	40.21[15.35,76.63]	19.26[10.72,29.24]	−2.25[−2.62,–1.88]	61.16[26.89,116.25]	4.72[0.72,16.96]	−8.01[−8.66,–7.36]	8.89[1.96,16.67]	13.86[3.05,24.52]	1.59[−0.94,4.19]	15.11[11,18.99]	8.7[6.4,10.99]	−1.58[−3.19,0.06]	−0.23[−0.93,1.12]	0[−0.34,0.72]	NS
Andean Latin America	49.49[23.84,79.4]	21.9[13.57,33.55]	−2.47[−2.99,–1.94]	37.76[17.31,65.39]	5.28[0.88,17.63]	−6.17[−6.71,–5.63]	2.77[0.54,5.65]	4.8[0.96,9.56]	2.18[−1.52,6.02]	13[10.27,15.98]	8.82[6.45,11.73]	−1.04[−1.28,–0.8]	−0.16[−0.32,–0.07]	−0.03[−0.14,0.14]	NS
Central Latin America	64.4[36.65,100.15]	25.34[17.78,33.9]	−2.8[−3.39,–2.2]	34.56[12.85,76.21]	9.43[2.77,29.43]	−4.28[−4.88,–3.68]	32.32[7.44,53.77]	10.67[2.34,19.17]	−3.46[−4.88,–2]	20.88[17.58,23.42]	13.7[11.18,15.71]	−1.64[−1.84,–1.45]	−0.14[−0.57,0.45]	0.41[−0.06,1.12]	NS
Southern Latin America	31.96[16.53,56.46]	22.06[12.43,34.77]	−1.15[−2.04,–0.25]	13.6[2.97,35.54]	0.6[0,5.85]	−9.72[−10.35,–9.1]	6.12[1.22,12.29]	6.19[1.2,12.21]	0.16[−0.14,0.46]	24.02[20.15,27.16]	19.53[16.67,21.96]	−0.41[−0.85,0.03]	0.16[−0.37,0.89]	0.11[−0.38,0.8]	1.77[0.35,3.22]
Central Sub-Saharan Africa	31.16[16.24,53.65]	27.36[15.88,42.89]	−0.42[−0.63,–0.21]	234.16[149.36,346.48]	177.01[101.68,299.98]	−0.91[−1.04,–0.78]	29.42[4.88,58.13]	33.96[5.79,70.46]	0.22[−2.22,2.71]	9[5.23,14.27]	6.41[3.75,10.7]	−1.49[−1.79,–1.19]	−1.72[−6.05,–0.47]	−0.87[−2.2,0.29]	NS
Eastern Sub-Saharan Africa	17.47[10.84,25.72]	15.44[9.51,23.55]	−0.31[−0.7,0.08]	201.28[150.02,246.48]	138.34[104.55,172.65]	−1.21[−1.3,–1.11]	10.07[1.87,19.04]	17.07[3.34,31.3]	1.71[−1.58,5.11]	18.35[12.8,23.41]	11.92[9.01,15.2]	−1.58[−2.05,–1.11]	0.25[−0.47,1.08]	0.44[−0.13,1.43]	NS
Western Sub-Saharan Africa	35.35[20.66,53.87]	30.72[17.96,46.22]	−0.47[−1.04,0.11]	111.75[80.29,142.7]	75.89[52.77,102.48]	−1.26[−1.47,–1.04]	14.46[2.78,26.51]	19.99[4.24,35.42]	0.96[−1.39,3.36]	2.1[0.51,3.94]	1.02[0.28,1.87]	−2[−2.62,–1.38]	3.35[1.46,6.39]	4.05[2.14,7.29]	1.37[0.86,1.88]
Southern Sub-Saharan Africa	46.29[32.1,62.57]	43.22[30.94,56.71]	−0.23[−0.89,0.42]	70.64[36.43,115.98]	27.59[14.16,53.05]	−2.98[−3.33,–2.63]	8.24[1.54,15.3]	15.58[3.21,27.93]	1.93[−1.89,5.9]	29.34[23.53,37.13]	26.36[21.8,30.98]	−0.4[−0.93,0.12]	−0.03[−0.69,0.91]	0.06[−0.6,0.96]	NS
North Africa and Middle East	60.43[42.32,79.64]	50.83[39.2,63.95]	−0.71[−1.22,–0.2]	37.61[19.58,69.26]	7.47[4.91,11.27]	−5.09[−5.17,–5]	32.65[6.24,59.79]	25.61[5.42,45.34]	−0.72[−1.35,–0.08]	27.32[19.5,34.88]	19.68[15.27,23.99]	−1.04[−1.41,–0.67]	2.92[−0.76,8.37]	3.32[−0.26,8.47]	0.16[−0.18,0.5]
Australasia	9.68[0.3,27.63]	8.26[4.78,12.05]	−0.95[−2.79,0.92]	0.2[0,1.9]	0.01[0,0.04]	−10.26[−10.99,–9.52]	2.27[0.37,5.38]	2.43[0.42,5.25]	0.41[−0.52,1.35]	24.43[20.18,27.79]	15.66[13.01,18.01]	−1.55[−2.01,–1.09]	0.19[−0.49,1.1]	0.11[−0.23,0.63]	0.62[−0.49,1.73]
High-income Asia Pacific	14.63[4.09,29.92]	7.03[4.01,10.65]	−2.46[−2.85,–2.08]	0.27[0.02,1.59]	0[0,0.02]	−13.98[−14.53,–13.43]	6.74[1.43,12.06]	6.03[1.28,10.68]	−0.41[−1.85,1.05]	13.79[11.66,15.42]	6.43[5.22,7.41]	−2.57[−3.28,–1.85]	0.23[−0.55,1.12]	0.12[−0.26,0.6]	NS
Caribbean	16.21[6.58,32.82]	19.6[9.77,33.15]	0.72[0.17,1.27]	26.91[13.84,41.39]	16.72[7.33,27.73]	−1.52[−1.71,–1.32]	5.63[1,10.71]	4.13[0.72,8.05]	−0.97[−3.38,1.5]	1.12[0.42,1.81]	1.11[0.47,1.71]	−0.54[−1.7,0.64]	−0.14[−0.39,−0.03]	–0.03[−0.17,0.2]	NS
Oceania	61.38[16.87,153.81]	64.98[22.27,153.15]	0.23[−0.01,0.47]	638.8[442.33,857.03]	485.81[331.47,667.02]	−0.88[−0.96,–0.8]	3.1[0.48,7.39]	2.06[0.41,4.02]	−2.45[−3.22,–1.66]	56.91[39.18,76.69]	45.22[32.87,59.23]	−0.78[−0.96,–0.59]	−0.61[−2.31,0.62]	−0.38[−1.46,0.63]	NS

**Table 2:** Age-standardized DALY rates (ASDR) and average annual percent change (AAPC) of COPD in older adults attributable to environmental factors across global, SDI, and GBD regions, 1990–2021.

Location	Ambient particulate matter pollution			Household air pollution from solid fuels			Ambient ozone pollution			Low temperature			High temperature		
	1990	2021	AAPC	1990	2021	AAPC	1990	2021	AAPC	1990	2021	AAPC	1990	2021	AAPC
Global	1384.69[919.53,2023.59]	1364.67[981.69,1687.82]	−0.23[−0.65,0.2]	4181.29[3306.4,4993.5]	1134.03[675.61,1903.06]	−4.19[−4.39,–3.98]	815.6[178.21,1434.12]	694.12[151.79,1190.36]	−0.5[−0.92,–0.08]	838.22[700.06,983.87]	411.38[324.46,504.24]	−2.38[−3.26,–1.49]	49.08[−22.1,161.32]	53.3[−10.54,146.6]	0.74[0.23,1.24]
High SDI	616.95[406.75,880.1]	255.12[176.88,339.4]	−2.83[−3.1,–2.56]	113.82[41.77,238.07]	0.93[0,8.07]	−14.45[−14.87,–14.02]	241.11[51.84,418.31]	165.15[36.01,287.22]	−1.17[−1.69,–0.64]	372.73[328.44,409.61]	262.82[230.2,288.16]	−1.45[−1.57,–1.33]	6.33[−13.32,32.03]	8.09[−9.75,33.44]	1.2[0.49,1.91]
High-middle SDI	1747.56[1089.58,2688.58]	1193.66[917.1,1492.22]	−1.31[−1.57,–1.05]	3733.37[2658.37,4753.91]	92.63[3.72,561.62]	−11.38[−12.24,–10.51]	882.01[188.31,1539.54]	399.49[81.93,706.58]	−2.46[−3.01,–1.9]	1086.21[924.9,1246.42]	425.74[348.55,509.24]	−3.01[−3.8,–2.21]	35.67[−30.95,137.24]	20.25[−18.94,74.69]	−1.94[−2.59,–1.27]
Middle SDI	2109.54[1237.94,3375.26]	2067.27[1460.05,2555.67]	−0.28[−1,0.44]	8311.41[6539.39,9884.74]	775.18[157.12,2187.76]	−7.48[−8.29,–6.66]	1314.28[282.35,2301.68]	663.66[145.57,1151.1]	−2.23[−2.96,–1.5]	1534.78[1262.51,1811.52]	557.3[440.54,688.71]	−3.25[−4.41,–2.07]	51.77[−41.85,184.91]	45.29[−9.68,123.2]	−0.31[−0.88,0.26]
Low-middle SDI	1463.93[858.85,2268.11]	2405.51[1404.31,3324.12]	1.61[0.86,2.36]	7341.6[5759.15,9041.8]	4416.19[2853.85,6234.13]	−1.61[−2.04,–1.18]	1183.13[229.22,2155.52]	1975.43[429.55,3352.97]	1.85[1.11,2.6]	493.77[244.09,803.63]	416.56[190.49,677.69]	−0.48[−0.77,–0.2]	160.99[−16.69,442.74]	192.4[−12.32,479.08]	1.15[0.58,1.71]
Low SDI	1173.61[699.62,1794.51]	1448.66[929,2055.62]	0.57[−0.06,1.21]	6455.29[5036.51,7967.87]	5304.95[4174.15,6444.15]	−0.59[−0.81,–0.37]	1096.87[210.2,2010.72]	1613.36[341.56,2777.93]	1.34[0.38,2.31]	463.75[267.26,693.51]	389.13[219.52,583.04]	−0.8[−2.53,0.95]	92.71[−23.45,282.08]	113.54[−22.19,312.13]	0.06[−2.61,2.8]
Western Europe	611.96[281.59,1049.35]	196.56[132.96,266.21]	−3.72[−3.92,–3.51]	3.77[0.03,31.72]	0.13[0,1.09]	−10.32[−10.98,–9.66]	193.88[42.77,338.7]	139.33[30.63,243.18]	−0.99[−2.31,0.35]	369.35[322.86,420.96]	260.03[224.75,297.75]	−1.41[−1.73,–1.09]	0.23[−7.22,7.49]	1.09[−3.65,6.47]	NS
Central Europe	944.14[519.99,1481.33]	372.25[288.61,466.72]	−3[−3.32,–2.67]	459.77[119.89,1362.02]	30.99[0.78,228.12]	−8.44[−9.11,–7.77]	365.88[80.2,646.57]	123.2[26.49,220.55]	−3.79[−5.08,–2.48]	456.36[402.37,515.07]	254.29[220.68,286.2]	−2.29[−2.64,–1.94]	0.72[−16.57,20.22]	4.33[−10.59,22.92]	NS
Eastern Europe	1180.65[525.63,1936.94]	183.63[112.3,269.68]	−5.78[−6.49,–5.07]	115.8[27.31,448.46]	9.12[1.18,45.12]	−7.94[−8.94,–6.93]	387.54[80.31,688.74]	53.68[11.02,98.12]	−6.18[−6.89,–5.47]	520.02[459.59,556.41]	167.21[139.35,192.9]	−3.73[−4.66,–2.79]	−0.75[−11.01,9.01]	2.21[−5.36,9.51]	NS
East Asia	3620.07[1709.54,6512.6]	2543.03[1821.55,3219.73]	−1.19[−1.41,–0.98]	14425.5[10996.48,17501.45]	812.89[208.82,2460.74]	−8.96[−9.81,–8.1]	2395.5[506.43,4201.66]	738.91[152.35,1325.66]	−3.83[−4.41,–3.25]	2988.53[2445.8,3509.32]	862.74[686.05,1061.38]	−4.16[−5.33,–2.98]	99.22[−90.78,377.86]	40.55[−31.91,142.06]	−3.03[−3.82,–2.24]
Central Asia	814[373.32,1504.82]	636.58[446.26,832.58]	−0.8[−1.76,0.17]	947.78[419.93,1821.02]	185.11[85.85,396.89]	−5.13[−5.54,–4.72]	539.61[107.32,942.18]	262.84[50.85,488.7]	−2[−2.75,–1.24]	542.24[468.57,601.68]	317.05[270.93,366.57]	−1.71[−2.83,–0.58]	12.81[−22.2,50.75]	20.05[−10.55,54.69]	1.84[−1.04,4.8]
South Asia	2001.62[1065.07,3252.44]	3404.55[2184.16,4506.08]	1.61[0.7,2.52]	9167.1[7061.6,11469.76]	4930.75[3264.05,6956.34]	−2.05[−2.42,–1.69]	1691.91[323.39,3089.17]	2664.09[578.19,4523.65]	1.55[0.87,2.24]	587.97[229.47,1025.46]	504.15[192.77,851.05]	−0.43[−0.79,–0.08]	219.35[−37.92,612.48]	237.57[−28.77,604.36]	0.83[0.24,1.43]
Southeast Asia	1010.49[450.07,1832.37]	1040.73[703.84,1370.61]	0.07[−0.17,0.31]	4226.27[3130.43,5304.99]	1189.27[482.85,2263.42]	−4.07[−4.29,–3.85]	148.34[27.89,296.74]	307.27[61.37,559.66]	2.4[−1.61,6.56]	103.44[50.43,164.14]	62.79[34.38,94.25]	−2.1[−2.5,–1.69]	19.52[−4.73,42.23]	32.64[16.42,61.21]	NS
High-income North America	495.31[178.49,891.38]	172.85[82.28,276.22]	−3.2[−3.91,–2.49]	0.38[0,2.69]	0.07[0,0.39]	−5.31[−5.61,–5.01]	360.45[79.44,624.34]	241.07[52.51,420.75]	−1.18[−1.59,–0.76]	385.25[328.44,423.21]	388.84[332.83,429.73]	−0.04[−0.34,0.26]	8.16[−26.43,49.94]	16.38[−21.57,72.66]	2.52[1.4,3.66]
Tropical Latin America	695.23[263.48,1324.28]	351.03[199,536.83]	−2.11[−2.51,–1.72]	1017.69[444.71,1945.6]	84.69[12.81,301.2]	−7.79[−8.25,–7.32]	142.61[31.47,267.65]	220.9[48.48,390.94]	1.74[−0.68,4.23]	245.84[180.02,307.34]	138.97[102.87,174.71]	−1.63[−3.25,0.03]	−3.57[−15.17,18.48]	0.02[−5.51,11.5]	NS
Andean Latin America	822.81[397.09,1314.72]	377.03[238.56,560.68]	−2.34[−2.92,–1.75]	639.96[296.24,1090.35]	92.44[15.81,303.81]	−6.09[−6.54,–5.64]	41.51[7.97,84.72]	67.05[13.5,134.03]	1.9[−1.67,5.6]	190.79[150.23,234.16]	121.37[87.95,161.9]	−1.32[−1.54,–1.1]	−2.3[−4.58,–0.91]	−0.31[−2.03,2.15]	NS
Central Latin America	1046.94[593.76,1638.74]	426.23[302.33,567.49]	−2.71[−3.18,–2.23]	580.19[218.9,1255.26]	165.16[50.12,496.26]	−4.04[−4.32,–3.76]	460.37[105.68,765.33]	152.42[33.34,273.53]	−3.48[−4.97,–1.96]	304.47[256.15,341.1]	192.76[158.17,221.04]	−1.72[−2.09,–1.35]	−2.15[−8.57,6.21]	5.97[−1.02,16.41]	NS
Southern Latin America	559.79[289.42,979.91]	371.98[210.47,585.77]	−1.18[−1.7,–0.66]	235.49[51.13,614.9]	9.84[0.03,95.61]	−9.83[−10.43,–9.23]	97.24[19.28,195.85]	94.3[18,186.74]	−0.01[−0.28,0.27]	382.49[322.1,431.96]	294.82[254.36,331.44]	−0.62[−1.04,–0.2]	2.63[−5.92,14.24]	1.7[−5.86,12.51]	1.68[0.28,3.11]
Central Sub-Saharan Africa	581.06[310.13,984.44]	541.36[326.36,832.82]	−0.23[−0.49,0.02]	4443.91[2976.06,6295.62]	3435.08[2165.11,5349.65]	−0.84[−0.97,–0.72]	498.37[83.81,972.2]	564.16[96.82,1160.29]	0.15[−2.25,2.6]	151.36[87.92,239.07]	106.07[62.17,173.77]	−1.54[−1.85,–1.24]	−29.1[−102.53,–7.92]	−14.46[−36.45,4.89]	NS
Eastern Sub-Saharan Africa	334.02[212.24,490.75]	307.36[193.2,462.21]	−0.19[−0.58,0.2]	3855.18[2970.29,4643.23]	2752.7[2155.9,3348.96]	−1.1[−1.16,–1.04]	177.98[33.1,336.64]	282.03[55.85,517.31]	1.46[−1.81,4.85]	316.5[222.85,401.76]	195.45[148.16,248.38]	−1.76[−2.24,–1.29]	4.74[−7.49,18.85]	7.64[−1.96,24.49]	2.57[0.18,5.01]
Western Sub-Saharan Africa	692.92[410.65,1043.94]	642.06[373.78,957.46]	−0.26[−0.86,0.34]	2201.94[1644.4,2766.72]	1583.44[1117.47,2096.7]	−1.07[−1.23,–0.91]	240.1[46.47,441.43]	321.63[68.78,571.94]	0.85[−1.52,3.27]	35.03[8.33,65.79]	16.66[4.58,30.57]	−2.09[−2.72,–1.47]	58.14[25.87,110.3]	67.42[35.52,121.64]	1.22[0.71,1.73]
Southern Sub-Saharan Africa	899.57[629.01,1189.56]	899.8[659.08,1166.94]	0.04[−0.44,0.53]	1417.19[756.78,2253.76]	586.16[319.38,1093.95]	−2.84[−3.21,–2.47]	131.92[24.61,244.4]	258.58[53.53,462]	2.07[−1.7,5.98]	457.19[369.44,571.49]	432.18[360.01,505.54]	−0.19[−0.74,0.36]	−0.29[−10.98,14.92]	0.96[−9.98,15.86]	NS
North Africa and Middle East	1115.27[795.64,1452.92]	995.66[782.65,1232.6]	−0.5[−0.99,0]	715.45[396.24,1276.38]	145.2[97.04,213.26]	−5.03[−5.1,–4.97]	523.2[99.97,955.36]	385.93[82.03,682.46]	−0.92[−1.13,–0.72]	430.19[310.13,547.11]	291.16[226.46,354.72]	−1.3[−1.67,–0.93]	47.94[−12.06,136.92]	52.7[−3.39,133.57]	0.06[−0.29,0.4]
Australasia	183.59[5.56,521.03]	147.69[85.52,214.88]	−1.1[−2.85,0.68]	3.9[0,36.98]	0.14[0,0.83]	−10.37[−11.01,–9.73]	37.61[6.14,89.32]	36.24[6.36,78.05]	0.03[−0.91,0.97]	404.77[334.59,460.37]	233.62[196.11,267.48]	−1.91[−2.4,–1.42]	3.15[−8.14,18.21]	1.62[−3.34,9.31]	0.37[−1.87,2.65]
High-income Asia Pacific	281.85[77.17,570.91]	167.63[99.89,249.65]	−1.75[−2.02,–1.49]	5.17[0.34,29.44]	0.07[0,0.5]	−13.24[−13.74,–12.75]	96.96[20.74,173.39]	82.36[17.62,145.77]	−0.58[−2.03,0.9]	196.06[167.41,218.41]	86.53[71.59,99.54]	−2.75[−3.48,–2.02]	3.38[−8.02,16.43]	1.69[−3.62,8.26]	NS
Caribbean	297.61[119.92,602.97]	376.45[188.79,632.31]	0.86[0.37,1.35]	527.74[282.35,793.6]	345.18[161.82,562.86]	−1.34[−1.47,–1.21]	92.99[16.42,178.66]	70.55[12.23,137.62]	−0.86[−3.32,1.65]	17.69[6.69,28.65]	18.29[7.81,28.13]	−0.45[−1.62,0.74]	−2.55[−6.92,–0.48]	−0.67[−2.9,3.31]	NS
Oceania	1,077[291.12,2731.25]	1122.86[382.47,2637.84]	0.16[0.02,0.3]	11289.87[7937.14,15100.84]	8397.26[5774.13,11479.12]	−0.95[−1.02,–0.88]	45.91[7.22,107.29]	31.48[6.27,62.14]	−2.29[−3.1,–1.48]	973.22[667.77,1318.89]	741.43[535.57,976.96]	−0.89[−1.07,–0.71]	−10.05[−38.1,9.8]	−6.17[−23.62,10.13]	NS

Significant variations in the COPD burden due to environmental factors were observed across the five SDI regions. Specifically, low-middle SDI regions bore the highest burden of COPD attributable to environmental factors. In regions below middle SDI, household air pollution from solid fuels remained the dominant contributor to COPD burden as of 2021. In high SDI regions, the environmental burden of COPD was the lowest, with ambient particulate matter pollution being the leading contributor since the study’s outset, though its burden fell below that of low temperature in 2010. From middle to high-middle SDI regions, a similar transition in burden patterns was observed, where ambient particulate matter pollution replaced household air pollution from solid fuels as the primary contributor, with the crossover point occurring earlier as SDI increased. From 1990 to 2021, the ASMR and ASDR of COPD in older adults due to household air pollution from solid fuels and low temperature decreased across all SDI regions (AAPC < 0, *p* < 0.05). However, the burden from ambient particulate matter pollution and ambient ozone pollution increased in regions below middle SDI (AAPC > 0, *p* < 0.05). High temperature contributed the lowest burden among environmental factors across all SDI regions, but notably, it exhibited an increasing trend in high SDI regions (AAPC > 0, *p* < 0.05).

The analysis of trends and burden of COPD in older adults due to environmental factors was extended to 21 GBD-defined geographical regions (Figures 1, 2 and Tables 1, 2). In 2021, for ambient particulate matter pollution, the highest ASDR burden was observed in South Asia (3404.55 [2184.16, 4506.08] per 100,000 population) and East Asia (2543.03 [1821.55, 3219.73] per 100,000 population). Moreover, while the burden decreased in most regions from 1990 to 2021, South Asia exhibited the largest increase in ASDR (1.61 [0.7, 2.52]). The ASDR of COPD due to household air pollution from solid fuels decreased across all regions from 1990 to 2021; however, as of 2021, several regions still faced substantial burdens, such as Oceania (8397.26 [5774.13, 11479.12]), South Asia (4930.75 [3264.05, 6956.34]), and Central Sub-Saharan Africa (3435.08 [2165.11, 5349.65]). In contrast, regions like High-income North America, Western Europe, Australasia, and High-income Asia Pacific had ASDR values attributable to household air pollution from solid fuels below 1, highlighting a stark disparity with high-burden regions. The burden of COPD in older adults due to ambient ozone pollution increased in half of the regions, primarily in Asia, Latin America, and Africa, with South Asia (2664.09 [578.19, 4523.65]) and East Asia (738.91 [152.35, 1325.66]) exhibiting the highest ASDR, far exceeding other regions. The burden due to low temperature decreased across all regions from 1990 to 2021, with East Asia recording the highest ASDR in 2021 (862.74 [686.05, 1061.38]), despite a significant decline since 1990. The estimation of trends and burden due to high temperature presented unique challenges. In some regions, insufficient data on high temperature led to unstable Bayesian simulation results, making meaningful trend analysis difficult when inputted into the Joinpoint model. Such cases were classified as NS (not significant). Based on available analyses, high temperature generally contributed a low burden to COPD across most regions but showed an increasing trend.

### 3.2 Trends and burden at the country level

**Figure 3:**
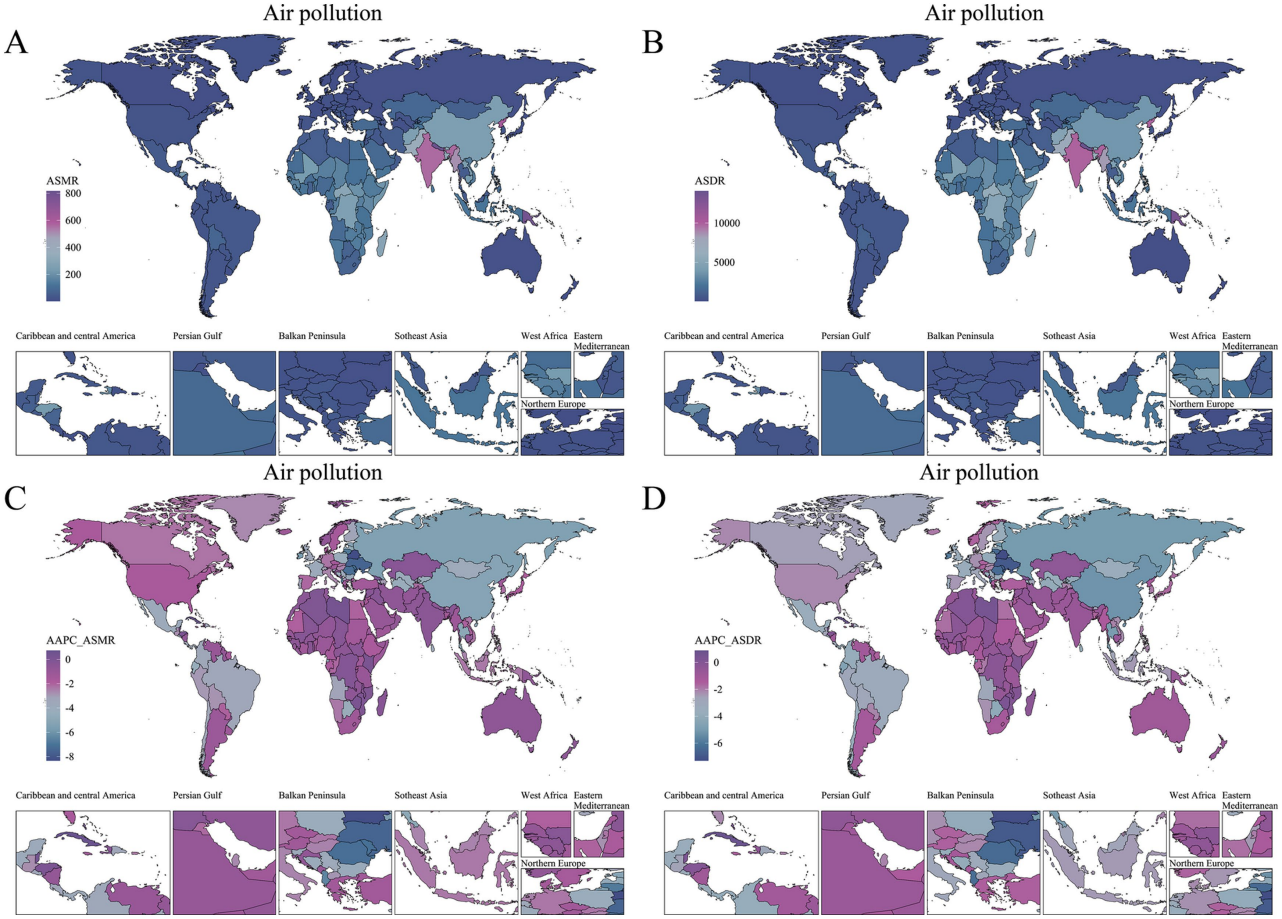
Global distribution of air pollution-related COPD burden and trends among older adults in 2021. **(A)** ASMR in 2021. **(B)** ASDR in 2021. **(C)** AAPC of ASMR (1990–2021). **(D)** AAPC of ASDR (1990–2021).

**Figure 4:**
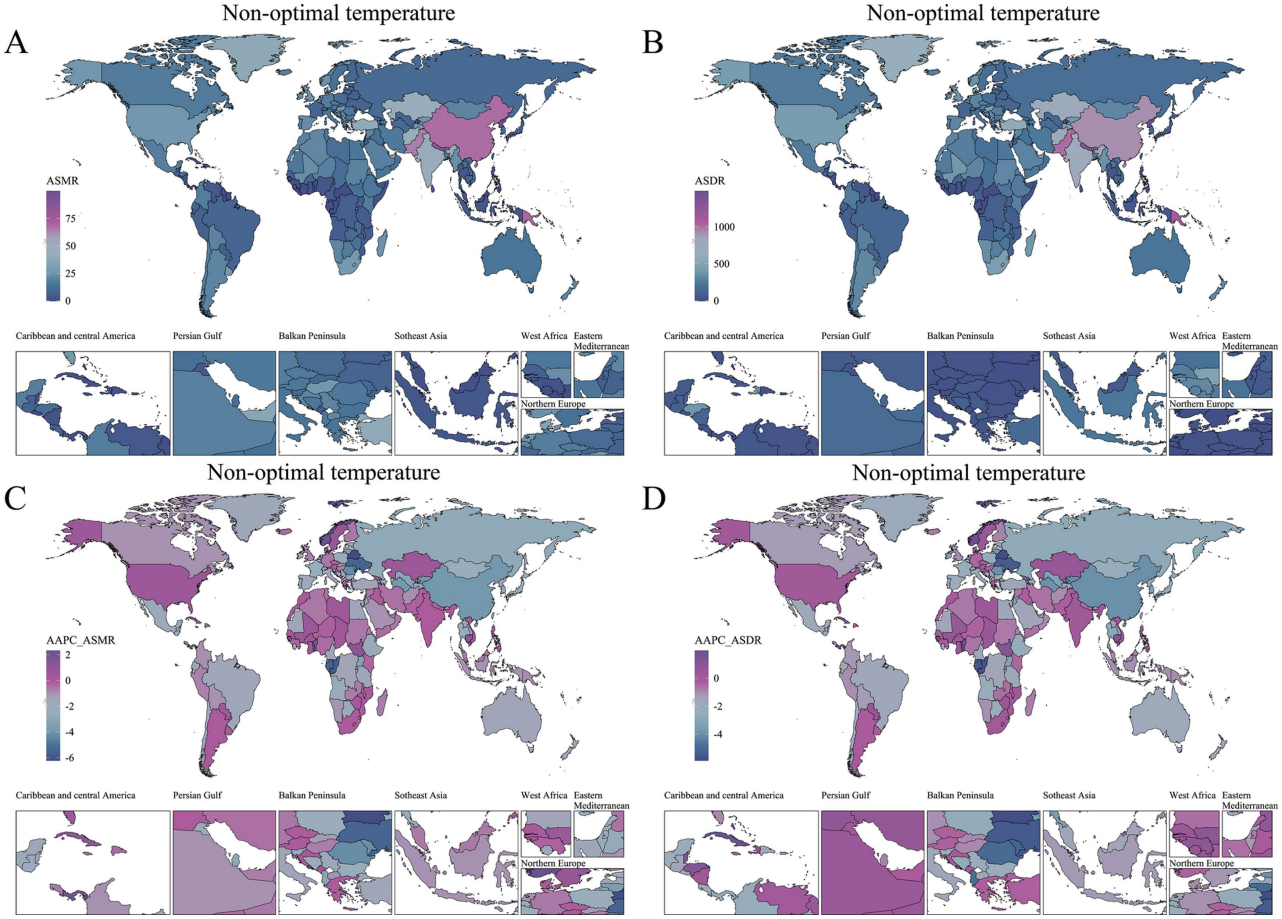
Global distribution of non-optimal temperature-related COPD burden and trends among older adults in 2021. **(A)** ASMR in 2021. **(B)** ASDR in 2021. **(C)** AAPC of ASMR (1990–2021). **(D)** AAPC of ASDR (1990–2021).

The analysis was further refined to the country level, using age-standardized rates and AAPC to quantify the COPD burden in older adults attributable to environmental factors (Supplementary Table). World map heatmaps were used to visualize the COPD burden due to air pollution and temperature issues across 204 countries and territories globally (Figures 3, 4). In 2021, the top three countries with the highest total DALYs due to air pollution-related COPD in older adults were Nepal (14076.33 [10601.33, 18394.69]) and India (9847.2 [7988.99, 11681.42]) from South Asia, and Papua New Guinea (13168.9 [9225.66, 17636.31]) from Oceania. Nepal and Papua New Guinea also ranked as the top two countries with the highest ASMR burden, with ASMR values exceeding 770, followed by North Korea (605.37 [395.82, 938.3]) from East Asia as the third highest. In 2021, extreme temperature-related COPD burden in older adults in North Korea, Nepal, Bhutan, and Papua New Guinea ranked among the top five globally, with ASMR values exceeding 68 and ASDR values exceeding 1,100. This geographical pattern mirrors that of the air pollution-related burden in older adults. Notably, China also experienced a substantial COPD burden in older adults due to extreme temperatures.

From 1990 to 2021, AAPC was used to quantify the trends in the COPD burden in older adults attributable to environmental factors across countries and regions (Figures 3, 4). Regarding the trend in air pollution-related COPD burden, only 12 out of 204 countries and territories exhibited an increasing trend (AAPC > 0), with most of these countries located in Caribbean Latin America and Africa. While the absolute burden of COPD due to non-optimal temperature was lower than that due to air pollution, its increasing trend was more pronounced. From 1990 to 2021, 28 countries and territories showed an increasing trend in COPD burden due to non-optimal temperature, with 13 countries from Africa and 6 from Asia being predominant.

### 3.3 Sex and age specific

**Figure 5:**
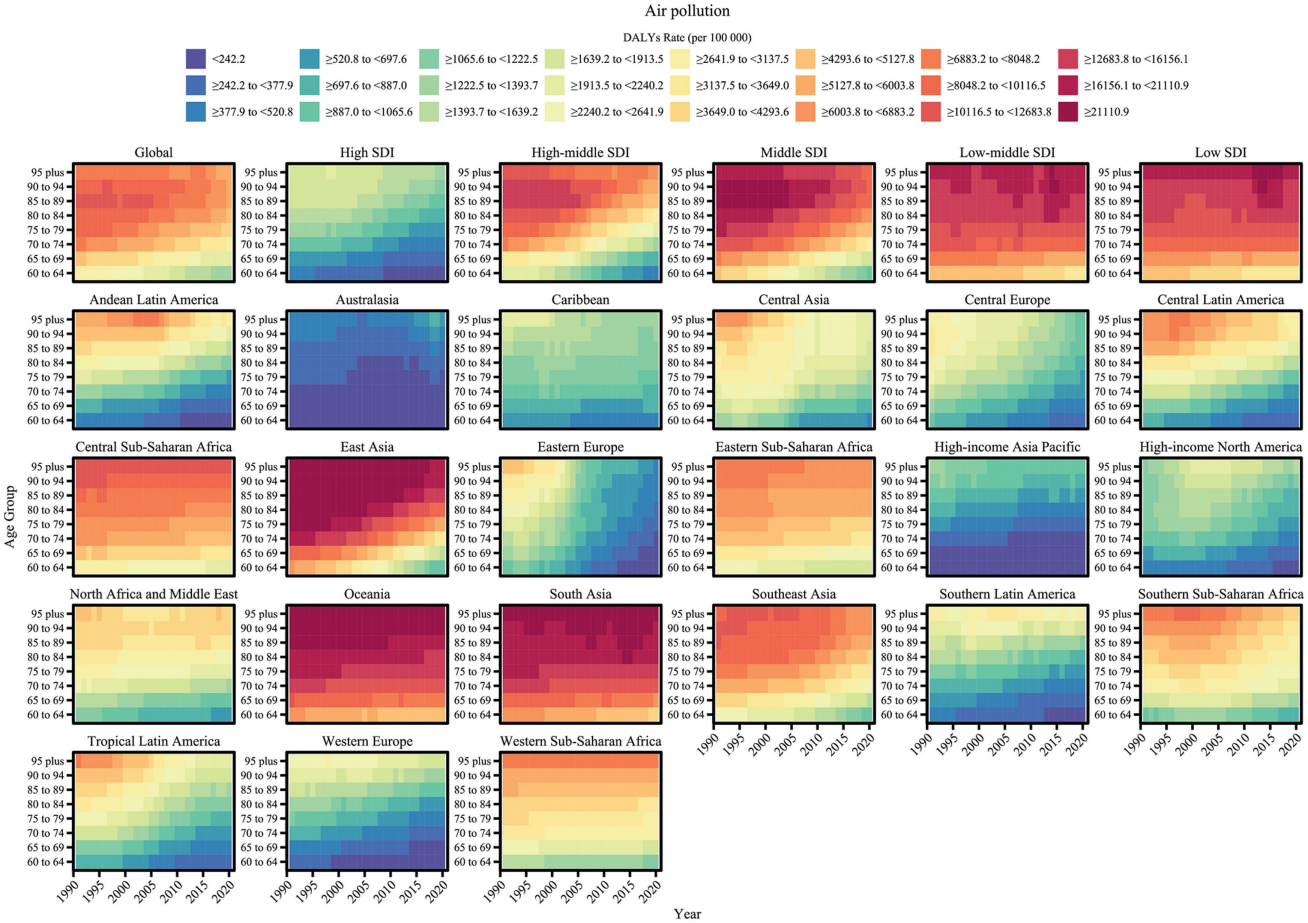
Age-specific trends in air pollution-related COPD burden (ASDR) among older adults by region, 1990–2021.

We examined the COPD burden in older adults due to environmental factors from 1990 to 2021 at global and regional levels, stratified by age and sex groups (Figure 5). In 2021, the global COPD burden due to air pollution was higher in males than in females (sex ratio > 1). A similar sex pattern was observed for the burden due to non-optimal temperature. Globally, the sex disparity in COPD burden due to both environmental factors decreased with increasing age, with the largest sex difference observed in the 65–90 age group. In regions with middle SDI and above, the sex difference in COPD burden due to air pollution was greater than in lower SDI regions. In contrast, the sex difference in COPD burden due to non-optimal temperature peaked in middle SDI regions. Across the 21 GBD regions, the sex differences in COPD burden due to air pollution and non-optimal temperature showed similar patterns, with the largest sex disparities observed in Eastern Europe and High-income Asia Pacific.

**Figure 6:**
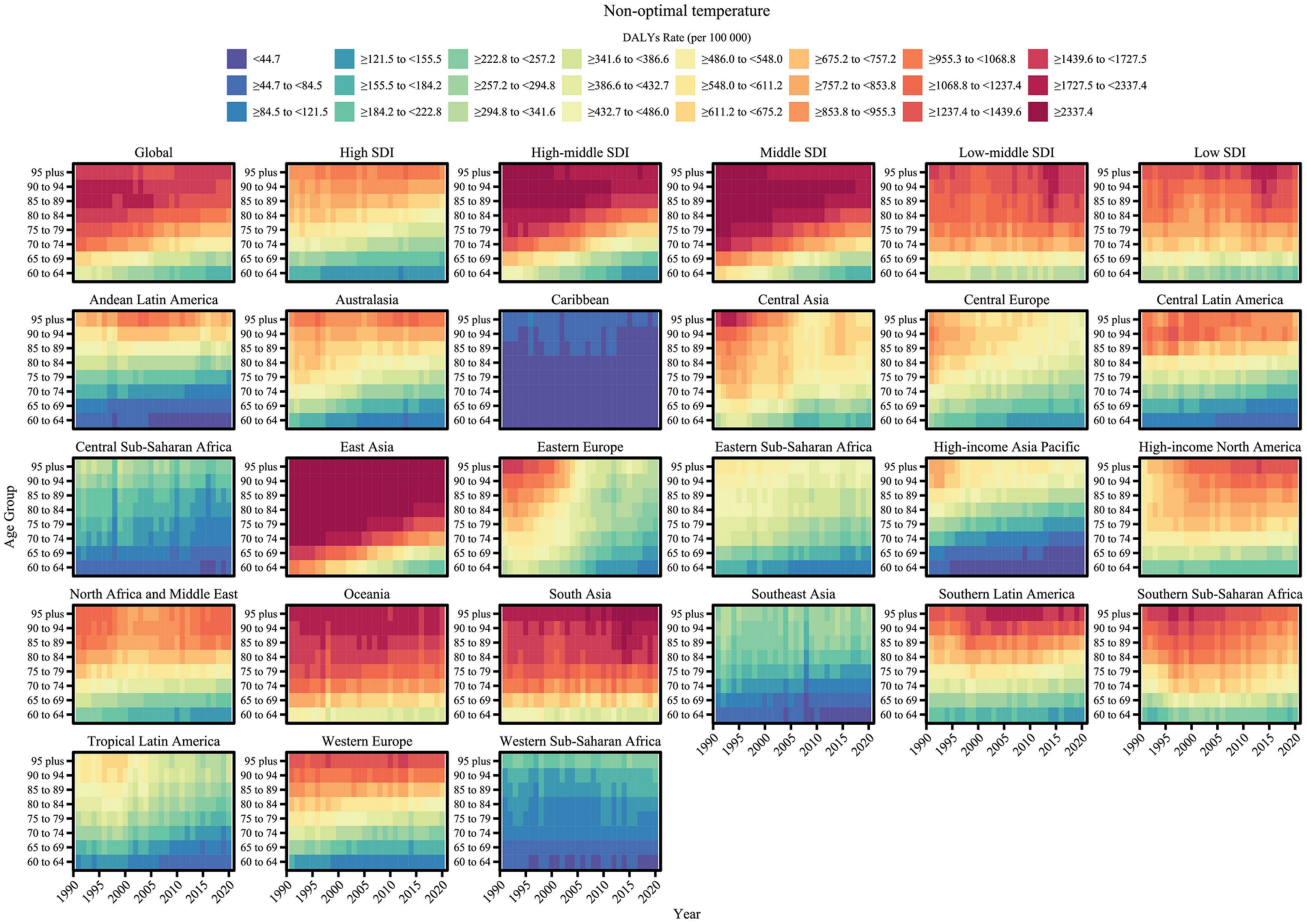
Age-specific trends in non-optimal temperature -related COPD burden (ASDR) among older adults by region, 1990–2021.

**Figure 7:**
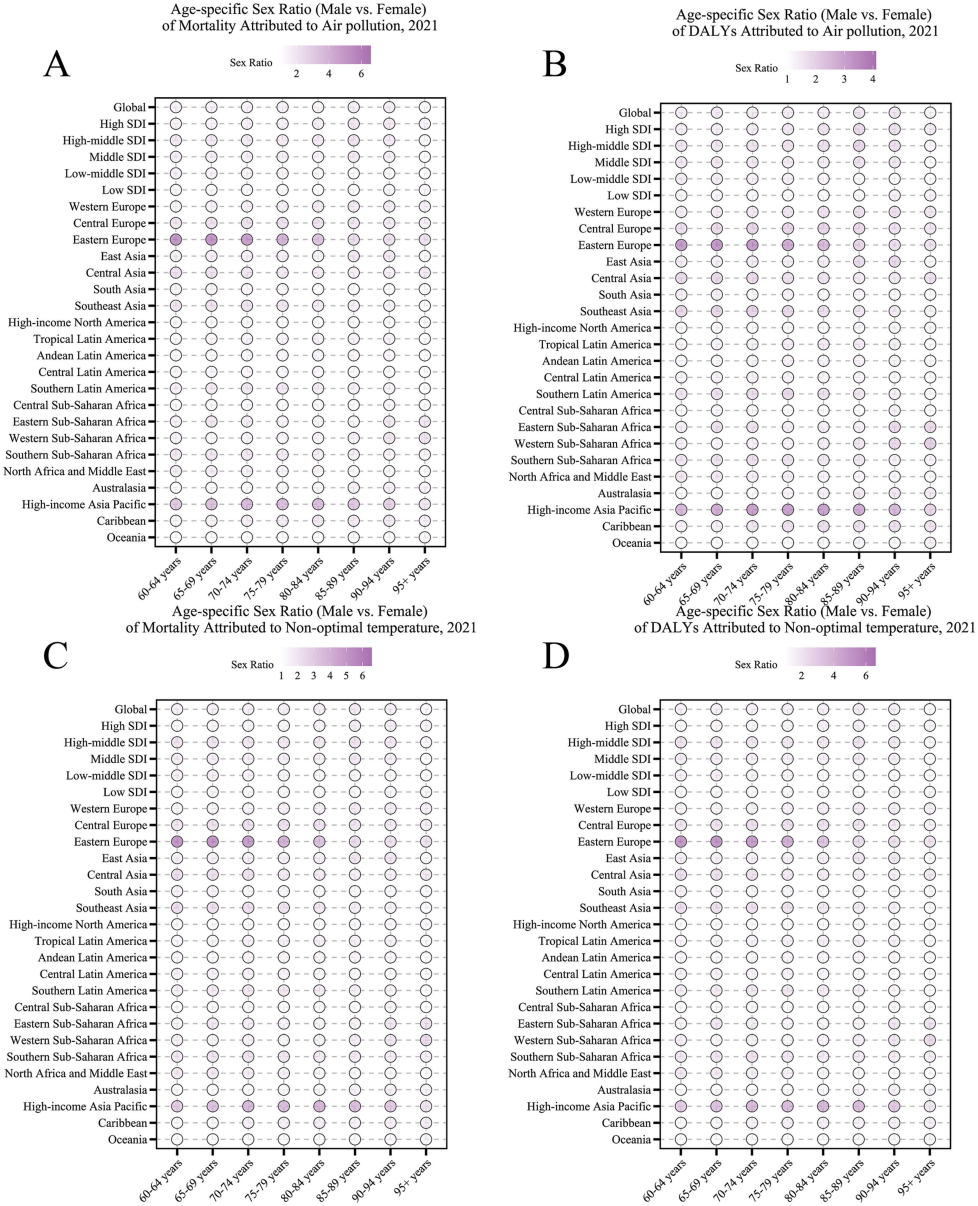
Age-specific sex ratios of COPD burden attributable to environmental factors among older adults in 2021. **(A)** Sex ratio (male vs. female) of mortality attributable to air pollution. **(B)** Sex ratio of DALYs attributable to air pollution. **(C)** Sex ratio of mortality attributable to non-optimal temperature. **(D)** Sex ratio of DALYs attributable to non-optimal temperature. Data are shown across age groups and regions (global, SDI levels, GBD regions), with color intensity indicating sex ratio magnitude.

Heatmaps were used to visualize the trends in COPD burden in older adults due to the two main environmental factors across detailed age groups from 1990 to 2021 at global and regional levels (Figures 6, 7). The age-specific patterns of COPD burden due to air pollution and non-optimal temperature were relatively similar. Specifically, the global COPD burden due to environmental factors increased with age, as reflected by rising ASMR and ASDR values. Across different SDI regions, the age patterns of the burden due to environmental factors were similar, with high SDI regions exhibiting the lowest burden. In low-middle SDI regions, the burden across all age groups showed no substantial relief over time. However, some differences were noted: for the burden due to non-optimal temperature, even in low-middle SDI regions, the lowest age group (e.g., 60–64 years) achieved a relatively low burden level, whereas for the burden due to air pollution in lower SDI regions, even younger age groups could not achieve a low burden level. Among the 21 GBD geographical regions, East Asia, South Asia, and Oceania bore the highest COPD burden in older adults due to environmental factors. In East Asia, the burden decreased significantly over time, reaching lower levels in younger age groups. However, in South Asia and Oceania, the COPD burden due to environmental factors remained high as of 2021. Furthermore, regarding regional differences in the burden due to air pollution and non-optimal temperature, non-optimal temperature contributed a notable burden in some regions with low air pollution burden. For instance, regions such as Australasia, Eastern Europe, High-income North America, Southern Latin America, and Western Sub-Saharan Africa, which had extremely low air pollution burdens, exhibited disproportionately high burdens due to non-optimal temperature.

### 3.4 Risk factor contributions

**Figure 8:**
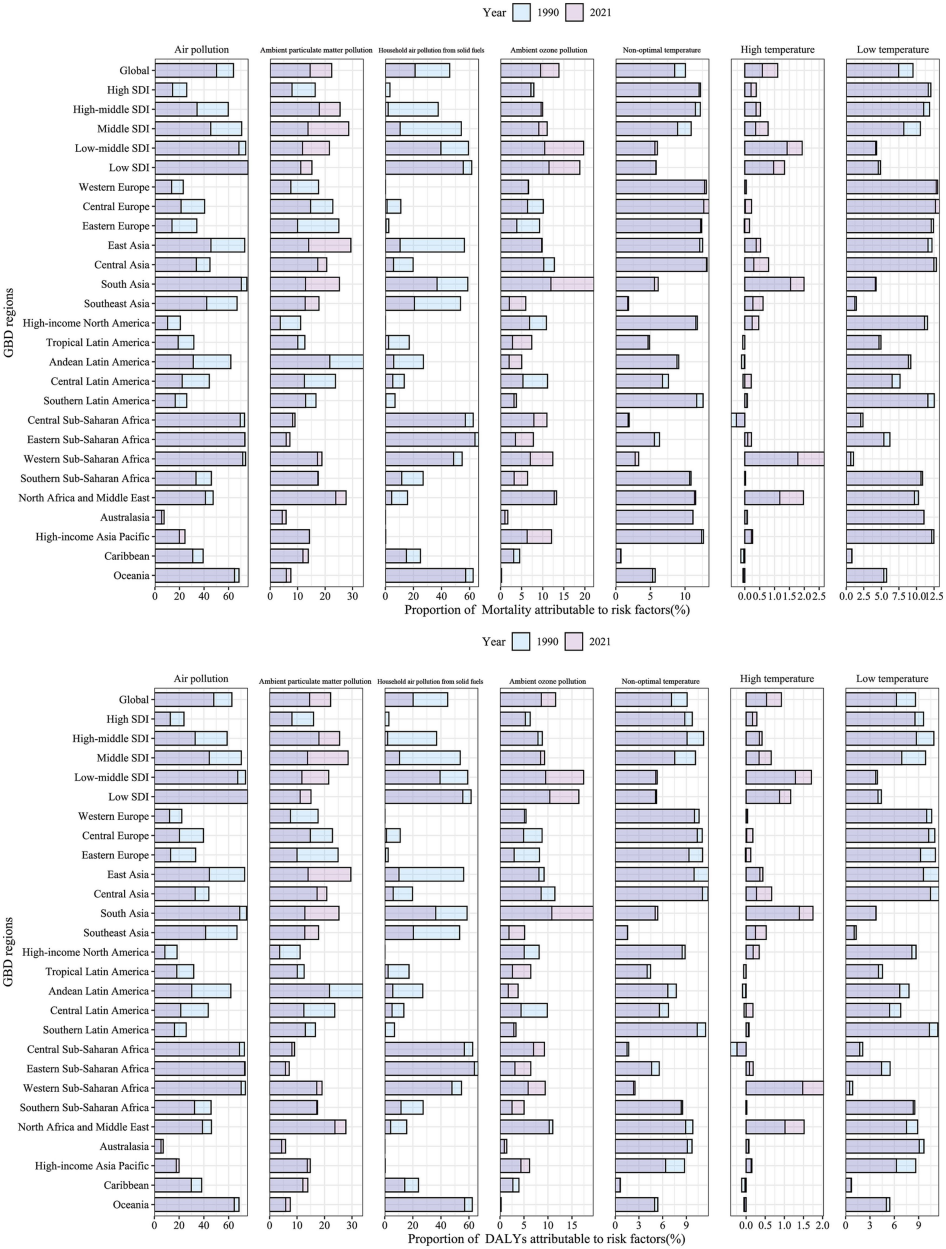
Proportion of COPD burden attributable to environmental risk factors among older adults in 1990 and 2021.

The population attributable fraction (PAF) was used to quantify the contribution of each environmental factor to the COPD burden in older adults, comparing 1990 and 2021. This analysis was extended to global, five SDI regions, and 21 GBD regions, with the burden decomposed into ASMR and ASDR (Figure 8). The attribution patterns of environmental factors to the COPD burden in older adults were similar for mortality and DALYs. In 2021, the global COPD burden attributable to air pollution accounted for 50% of the total, indicating that reducing global air pollution to the theoretical minimum risk exposure level (TMREL) could decrease the COPD burden in older adults by 50%. From 1990 to 2021, the PAF for air pollution-related COPD burden increased for ambient particulate matter pollution and ambient ozone pollution to 22 and 11%, respectively, while the PAF for household air pollution from solid fuels, despite a decline, remained high at 20%. Compared to 1990, the PAF for high temperature in 2021 increased but remained below 2%, whereas the PAF for low temperature, though decreased, still contributed 7.5%, making non-optimal temperature a dominant factor in COPD burden. The PAF for high temperature increased across all SDI regions. In high SDI regions, the PAF for all environmental factors except high temperature decreased, while in lower SDI regions, the PAF for ambient particulate matter pollution and ambient ozone pollution showed substantial increases. In 2021, among the 21 GBD regions, the PAF for ambient particulate matter pollution-related COPD increased in all Asian regions, with East Asia exhibiting the highest PAF. Although regions with increasing PAF for ambient ozone pollution were mostly in Latin America and Africa, South Asia showed the largest increase and had the highest PAF for ambient ozone pollution in 2021.

### 3.5 SDI-related analysis

**Figure 9:**
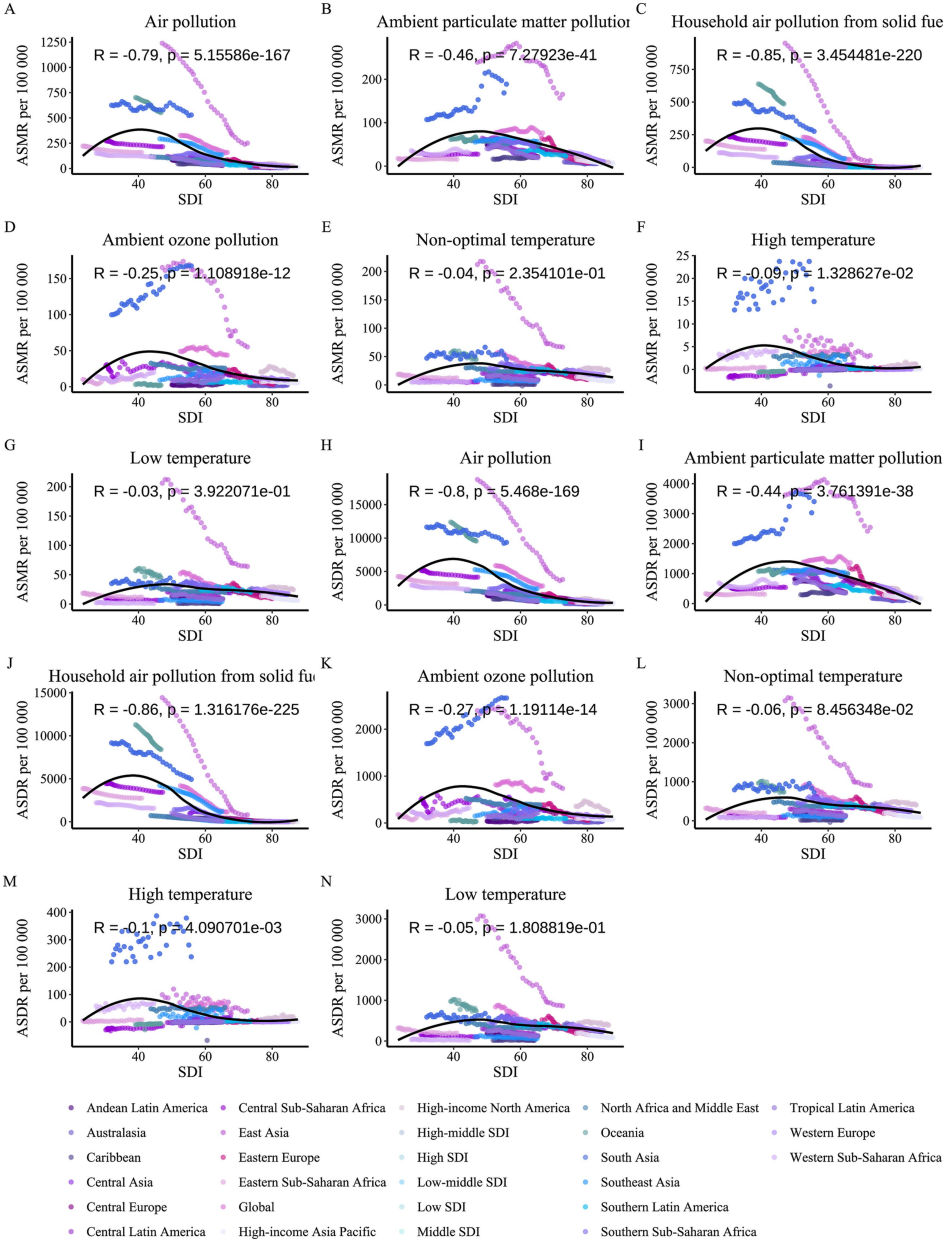
Correlation between SDI and COPD burden attributable to environmental factors among older adults, 1990–2021. **(A)** Air pollution vs. ASMR. **(B)** Ambient particulate matter pollution vs. ASMR. **(C)** Household air pollution from solid fuels vs. ASMR. **(D)** Ambient ozone pollution vs. ASMR. **(E)** Non-optimal temperature vs. ASMR. **(F)** High temperature vs. ASMR. **(G)** Low temperature vs. ASMR. **(H)** Air pollution vs. ASDR. **(I)** Ambient particulate matter pollution vs. ASDR. **(J)** Household air pollution from solid fuels vs. ASDR. **(K)** Ambient ozone pollution vs. ASDR. **(L)** Non-optimal temperature vs. ASDR. **(M)** High temperature vs. ASDR. **(N)** Low temperature vs. ASDR. Each panel plots SDI (*x*-axis) against ASMR or ASDR (*y*-axis), with data points colored by region, showing correlation coefficients (R) and *p*-values.

Analysis of trends in the COPD burden in older adults due to environmental factors revealed distinct burden patterns across different SDI regions. To further explore SDI-related burden patterns, we combined SDI scores for each region with burden levels from 1990 to 2021, conducting a correlation analysis between burden levels and SDI scores, followed by statistical testing (Figure 9). The results indicated a strong negative correlation between air pollution and the COPD burden in older adults (*R* < −0.79, *p* < 0.05), suggesting that as SDI decreases, the air pollution-related COPD burden in older adults increases. The strongest correlation was observed for household air pollution from solid fuels, indicating that low SDI regions struggle to control this burden effectively, while high SDI regions can manage it more successfully. The burden due to non-optimal temperature showed no significant correlation with SDI scores overall; however, a relatively weak but statistically significant negative correlation was observed between high temperature-related burden and SDI. In this analysis, we not only assessed the correlation between various environmental factors and the COPD burden in older adults with SDI but also fitted an average curve based on all included data, which can serve as a reference to identify outlier regions at the same SDI level. Overall, South Asia and East Asia exhibited significantly higher COPD burdens in older adults due to most environmental factors compared to other regions at similar SDI levels, suggesting that older populations in these regions may be more vulnerable to environmental factor-related COPD.

### 3.6 Relationship between burden and each risk factor

**Figure 10:**
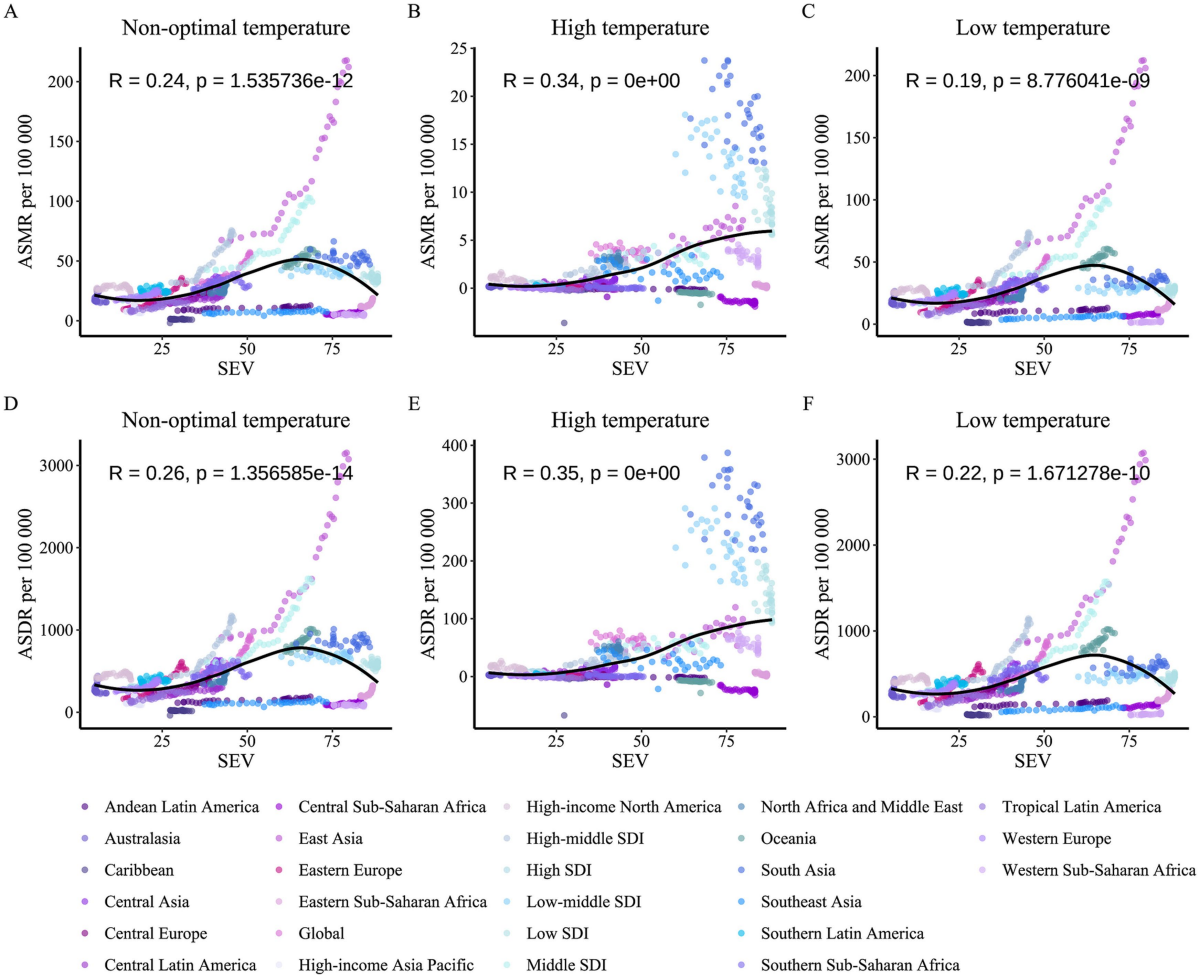
Correlation between air pollution SEV and COPD burden attributable to non-optimal temperature among older adults, 1990–2021. **(A)** Non-optimal temperature vs. ASMR per air pollution SEV. **(B)** High temperature vs. ASMR per air pollution SEV. **(C)** Low temperature vs. ASMR per air pollution SEV. **(D)** Non-optimal temperature vs. ASDR per air pollution SEV. **(E)** High temperature vs. ASDR per air pollution SEV. **(F)** Low temperature vs. ASDR per air pollution SEV. Each panel plots air pollution SEV (*x*-axis) against ASMR or ASDR (*y*-axis), with data points colored by region, showing correlation coefficients (R) and *p*-values.

**Figure 11:**
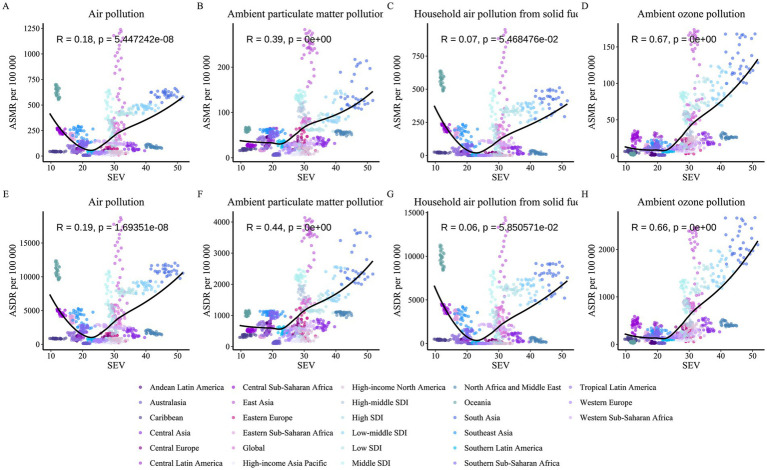
Correlation between non-optimal temperature SEV and COPD burden attributable to air pollution among older adults, 1990–2021. **(A)** Air pollution vs. ASMR per non-optimal temperature SEV. **(B)** Ambient particulate matter pollution vs. ASMR per non-optimal temperature SEV. **(C)** Household air pollution from solid fuels vs. ASMR per non-optimal temperature SEV. **(D)** Ambient ozone pollution vs. ASMR per non-optimal temperature SEV. **(E)** Air pollution vs. ASDR per non-optimal temperature SEV. **(F)** Ambient particulate matter pollution vs. ASDR per non-optimal temperature SEV. **(G)** Household air pollution from solid fuels vs. ASDR per non-optimal temperature SEV. **(H)** Ambient ozone pollution vs. ASDR per non-optimal temperature SEV. Each panel plots non-optimal temperature SEV (*x*-axis) against ASMR or ASDR (*y*-axis), with data points colored by region, showing correlation coefficients (R) and *p*-values.

Following a comprehensive analysis of the COPD burden in older adults due to the two main environmental factors, we observed substantial similarities between the burden patterns caused by air pollution and non-optimal temperature. Based on previous studies, we hypothesized a potential synergistic effect between air pollution and non-optimal temperature on the COPD burden in older adults. To test this hypothesis, we obtained standardized exposure values (SEV) and burden data for environmental factors across all regions from 1990 to 2021, conducting correlation analyses between air pollution SEV levels and the burden due to non-optimal temperature, and vice versa (Figures 10, 11). Interestingly, the results revealed that all non-optimal temperature factors, including high and low temperatures, were significantly positively correlated with regional air pollution SEV levels, with the correlation being particularly strong for high temperature and air pollution exposure levels. This suggests that as air pollution exposure levels increase, the COPD burden in older adults due to non-optimal temperature also rises. A similar trend was observed in the reverse analysis, where the burden due to air pollution showed a significant positive correlation with non-optimal temperature SEV levels, with the strongest correlation observed for the burden due to ambient ozone pollution. However, it is noteworthy that household air pollution from solid fuels did not exhibit a significant positive correlation with non-optimal temperature SEV levels (*p* > 0.05), indicating that household air pollution from solid fuels may be strongly correlated with SDI but lacks a significant association with non-optimal temperature factors.

## 4 Discussion

This study comprehensively evaluated the impact of environmental factors—specifically air pollution and non-optimal temperature—on the COPD burden in older adults from 1990 to 2021, elucidating trends, sex- and age-specific patterns, and the role of socioeconomic factors at global, regional, and national levels. A key finding is the stark regional disparity in COPD burden, with South Asia and East Asia bearing the heaviest burdens, particularly in low-SDI countries facing a persistent dual burden from ambient particulate matter (PM2.5) and household air pollution (ASDR in 2021: South Asia 3404.55 [2184.16, 4506.08], East Asia 2543.03 [1821.55, 3219.73] per 100,000). Globally, the COPD burden attributable to most environmental factors either declined or remained stable, reflecting progress in reducing household air pollution worldwide. However, the burden due to high temperature showed an increasing trend (AAPC > 0, *p* < 0.05), consistent with the exacerbation of climate change and the rising frequency of extreme heat events (20). High temperatures may aggravate COPD symptoms by inducing airway inflammation, reducing lung function, and increasing infection risks (21). Additionally, around 2015, ambient particulate matter pollution surpassed household air pollution to become the leading environmental contributor to the global COPD burden, highlighting new challenges driven by urbanization, industrialization, and increased traffic emissions (22). Regional analysis revealed that South Asia and East Asia bore the heaviest COPD burden, with low SDI countries facing a persistent dual burden from household air pollution and ambient particulate matter pollution.

Environmental factors influence COPD onset and exacerbation through various biological mechanisms (23). Air pollutants such as PM2.5, ozone, and household air pollution induce inflammation, oxidative stress, and airway hyperresponsiveness, disrupting the lung epithelial barrier and increasing infection risks (24–26). Non-optimal temperatures, particularly high and low temperatures, pose additional threats to COPD patients by directly irritating the airways, exacerbating inflammation, and impairing immune function (27–29). During high temperatures, elevated ozone levels further enhance the toxicity of PM2.5, while low temperatures increase airway sensitivity to pollutants, intensifying airway spasms and inflammatory responses (30, 31). Older adults are particularly susceptible to these environmental factors due to physiological deterioration, declining immune function, and the presence of comorbidities (32, 33), as evidenced in our study by the increasing COPD burden with age, with the heaviest burden observed in those over 95 years. These mechanisms explain the pronounced impact of environmental factors on COPD in older populations.

Sex- and age-specific analyses revealed heterogeneity in the COPD burden. Globally, males consistently exhibited a higher COPD burden than females, potentially due to greater exposure levels (e.g., occupational exposure) and physiological differences (34). However, the sex disparity diminished with increasing age, particularly in older age groups, suggesting increased vulnerability in females at advanced ages, possibly due to hormonal changes and a sharper decline in immune function (35). Additionally, sex disparity patterns varied across SDI regions, with middle-to-high SDI regions showing larger sex differences, reflecting the modulating role of socioeconomic factors on exposure patterns and health outcomes. Age patterns indicated that the COPD burden increased with age, particularly in low SDI regions, where even younger age groups failed to experience significant burden relief, underscoring the challenges in COPD prevention and control in these regions.

Correlation analysis between SDI and COPD burden revealed a negative association (*R* < −0.79, *p* < 0.05), indicating that high SDI regions effectively reduced the COPD burden through better environmental governance and healthcare resources. In contrast, South Asia and East Asia exhibited significantly higher burdens than other regions at similar SDI levels, highlighting their unique vulnerability to environmental health risks, likely due to high population density, industrial pollution, and inadequate policy implementation (36, 37). Analysis of the interaction between the two main environmental factors further revealed a synergistic effect: air pollution exposure levels were positively correlated with the COPD burden due to non-optimal temperature, and vice versa. This synergy may stem from co-exposure to ozone and PM2.5 under high temperatures or the enhanced irritative effects of pollutants on the airways during low temperatures, providing critical insights for integrated environmental management.

Despite offering a global-scale analysis, this study has limitations. First, the precision of GBD data in some regions is low, resulting in wide uncertainty intervals (UI) that affect the stability of estimates. Second, the Joinpoint model requires high data stability, and trend analysis for high temperature burden in some regions was inconclusive due to insufficient data. Additionally, this study only included risk factors available in the GBD database, potentially omitting other critical factors, which may lead to an underestimation of the complexity of the COPD burden. Future research should focus on refining regional analyses, exploring additional factors, and improving data quality. Meanwhile, policymakers should prioritize high-burden regions such as South Asia and East Asia, strengthening air pollution control, promoting clean energy, and developing health strategies to adapt to climate change, thereby alleviating the COPD burden in older adults.

## 5 Conclusion

This study elucidates the evolving environmental influences on the COPD burden in older adults from 1990 to 2021, revealing critical trends and interaction effects. While household air pollution has declined globally, ambient PM2.5 and high-temperature impacts have risen, disproportionately affecting low-SDI regions such as South and East Asia, where COPD burdens remain highest (e.g., South Asia ASDR: 3404.55 [2184.16, 4506.08] per 100,000). Notably, synergistic interactions between air pollution and temperature—particularly high temperatures amplifying PM2.5 and ozone toxicity through enhanced photochemical reactions and airway inflammation—exacerbate COPD severity. These findings underscore the urgent need for integrated health and environmental strategies that simultaneously address air quality and climate change, prioritizing vulnerable regions. Targeted interventions, such as stricter PM2.5 regulations, clean energy promotion, and climate-adaptive health policies, are essential to mitigate COPD burden and promote health equity in aging populations.

## Data Availability

The original contributions presented in the study are included in the article/Supplementary material, further inquiries can be directed to the corresponding author/s.
