# Supplementary figures and images for: Global burden of disease study on COPD in the older adult: comprehensive analysis of environmental factors and interaction effects
Source: Front Public Health. 2025 May 30;13:1597793. doi: 10.3389/fpubh.2025.1597793 (PMC12162481; doi:10.3389/fpubh.2025.1597793)

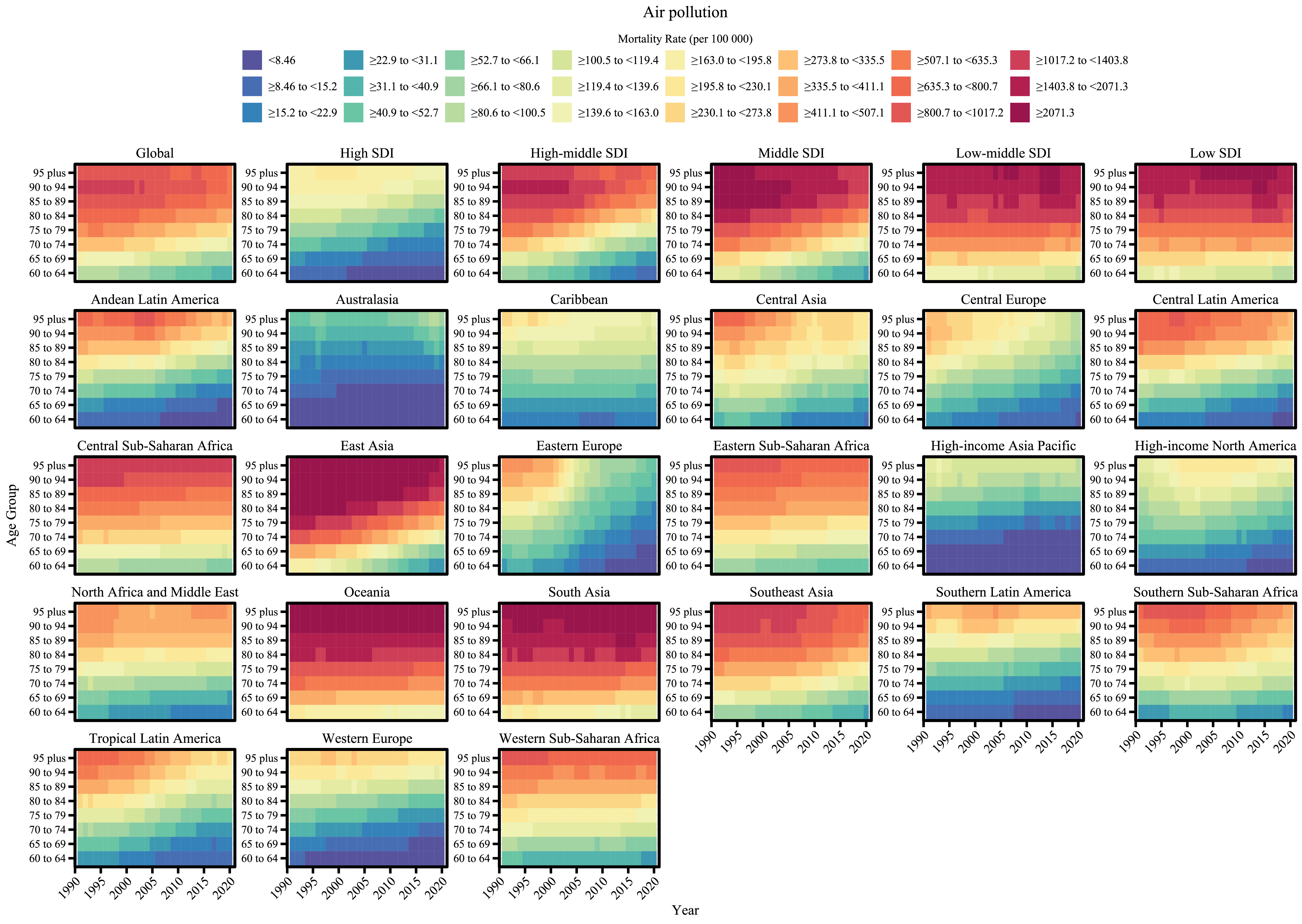

Supplement: Supplementary file 3 [file Data_Sheet_1.ZIP › supplement/FigureS1.jpg]

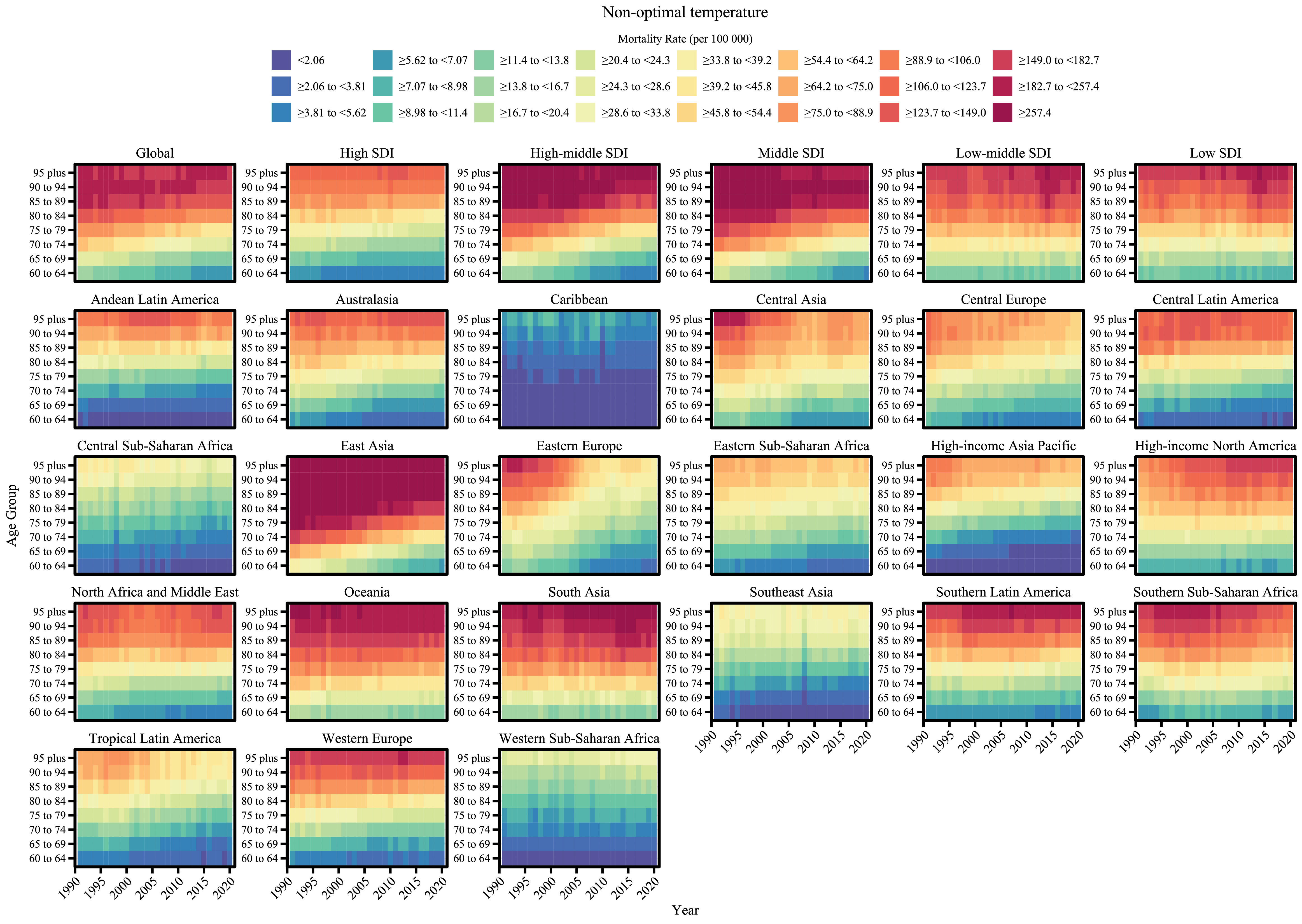

Supplement: Supplementary file 3 [file Data_Sheet_1.ZIP › supplement/FigureS2.jpg]
